# Cerebellar α1_D_- adrenergic receptors mediate stress-induced dystonia in tottering^tg/tg^ mice

**DOI:** 10.1007/s00018-025-05843-1

**Published:** 2025-10-06

**Authors:** Pauline Bohne, Mareike Josten, Lina Rambuscheck, Jana Brüggemann, Xinran Zhu, Max O. Rybarski, Melanie D. Mark

**Affiliations:** 1 Behavioral Neuroscience, Ruhr-University Bochum, ND7/31, Universitätsstr. 150, D-44780 Bochum, Germany; 2 Department of Zoology and Neurobiology, Ruhr-University Bochum, ND7/31, Universitätsstr. 150, D-44780 Bochum, Germany

**Keywords:** Dystonia, Adrenergic receptors, Episodic ataxia type 2, Cerebellum, Stress

## Abstract

Episodic ataxia type 2 (EA2) is an inherited neurological disorder, where patients suffer from chronic ataxia and severe episodes of motor dysfunction exhibited as dystonia. Despite other factors, physical and emotional stress triggers those episodes reliably in both human and mice. We used the well-established EA2 mouse model tottering to explore the cerebellar adrenergic receptor (AR) involvement in stress-induced dystonic attacks. We found that α1-ARs in cerebellar Purkinje cells (PCs) are activated by norepinephrine (NE), differentially expressed and required for initiation of dystonia, while α2-ARs are not. Moreover, pharmacological blockade and shRNA-induced knock down of cerebellar α1_D_-ARs was sufficient to effectively prevent stress-induced dystonia in homozygous tottering^tg/tg^ mice but had no impact on ataxia amelioration. In vivo recordings and live calcium (Ca^2+^) imaging of PCs demonstrated that α1_D_-AR blockade successfully protects PCs from NE-mediated erratic firing patterns through decreased release of Ca^2+^ from intracellular stores, thus preventing stress-induced dystonia. Together, our data show the modulatory effects of NE on dystonia severity and suggest a predominant role of cerebellar α1_D_-ARs in the formation of stress-induced dystonia in tottering^tg/tg^ mice.

**Supplementary Information:**

The online version contains supplementary material available at 10.1007/s00018-025-05843-1.

## Introduction

Episodic Ataxia Type 2 (EA2) is a rare, autosomal inherited episodic neurological disorder belonging to a broad family of channelopathies premised on mutations in ion channels. EA2 is caused by loss-of-function mutations in the CACNA1A gene, encoding the pore-forming α1 subunit of the P/Q-type voltage-gated calcium channel (Ca_V_2.1) [1], which is predominantly expressed on cerebellar Purkinje cells (PCs) and granule cells (GCs) and required for neurotransmitter release [2, 3]. EA2 patients suffer from permanent ataxia, nausea, nystagmus, cognitive deficits and episodes of increasing ataxia, instability and dystonia due to involuntary antagonistic muscle contraction [4]. Episodic attacks occur spontaneously or can be triggered by chemical stressors such as caffeine and ethanol, as well as by physical and psychological stressors [5]. Since these episodic attacks can last hours to days, patients are advised to remain relaxed by controlling their environment [6]. However, this is accompanied with lifetime limitations.

Similar to EA2 patients, tottering^tgtg^ mice carry a spontaneous loss-of-function mutation (P601L) in the CACNA1A gene, resulting in a ~ 40% reduction of Ca^2+^ currents through the P/Q-type channel [7], thereby resembling symptoms of EA2 patients including ataxia and episodes of severe paroxysmal nonkinesigenic dyskinesia. Stress, ethanol and caffeine reliably elicit attacks of motor dysfunction in tottering^tg/tg^ mice [8–12], making them an ideal animal model to study the neuronal basis of EA2 [13]. During stressful conditions, norepinephrine (NE) is released from the locus coeruleus (LC), innervating the whole brain. In the cerebellum, PCs are the main target of LC synapses [14], receiving predominantly inhibitory input through α- and β-adrenergic receptors (ARs) [15–17], resulting in long-lasting inhibition of PCs [18].

Tottering^tg/tg^ mice are hyperinnervated by NE [19], and pharmacological blockade of cerebellar α1-ARs was effective in preventing stress-induced dystonia [20], though the exact mechanism was not understood until recently. Snell and colleagues showed that NE-mediated stress-induced attacks of motor dysfunction are caused by avid burst firing of cerebellar PCs via activation of α1-ARs and consequent reduction of SK channel activity [21]. Consequently, cerebellar infusion of prazosin hydrochloride effectively prevented stress-induced attacks and the authors proposed α1-AR blockade as a potential treatment option. However, systemic administration of general α1-ARs antagonists targets ARs in the entire body, resulting in unwanted side effects. Here, we utilized the well-characterized EA2 mouse model tottering^tg/tg^ to identify a specific α1-AR subtype predominantly involved in formation of stress-mediated paroxysmal dyskinesia. We found that the α1_D_-AR specific antagonist BMY-7378 (BMY) successfully alleviated stress-induced dystonia and recovered PC simple spike (SS) firing after NE-mediated inhibition. Cerebellar α1_D_-ARs were also upregulated compared to tottering^−/−^ control mice. Additionally, chronic infusion of BMY and shRNA-induced knock-down of α1_D_-ARs in the cerebellar vermis of tottering^tg/tg^ mice reduced or prevented dystonic attacks and severity, verifying the cerebellar α1_D_-AR involvement in the formation of stress-induced paroxysmal dyskinesia. In vivo Ca^2+^ imaging of PCs using miniscopes showed an increase in intracellular calcium release at the onset of dystonic attacks, which was abolished after application of BMY-7378. These results indicate an impairment in the PC calcium homeostasis as the cause for dystonic episodes and strengthens the predominant involvement of PCs in dystonia [8, 22]. Our findings provide further insight into the noradrenergic modulation of the cerebellar circuitry in stress-induced paroxysmal dystonia in tottering^tg/tg^ mice. More importantly, we identified cerebellar α1_D_-ARs as the accountable adrenergic receptor for the formation of episodic dystonia.

## Results

### Upregulated α1_D_-adrenergic receptors located on cerebellar Purkinje cells mediate stress-induced paroxysmal dystonia in *tottering*^*tg/tg*^ mice

**Fig. 1:**
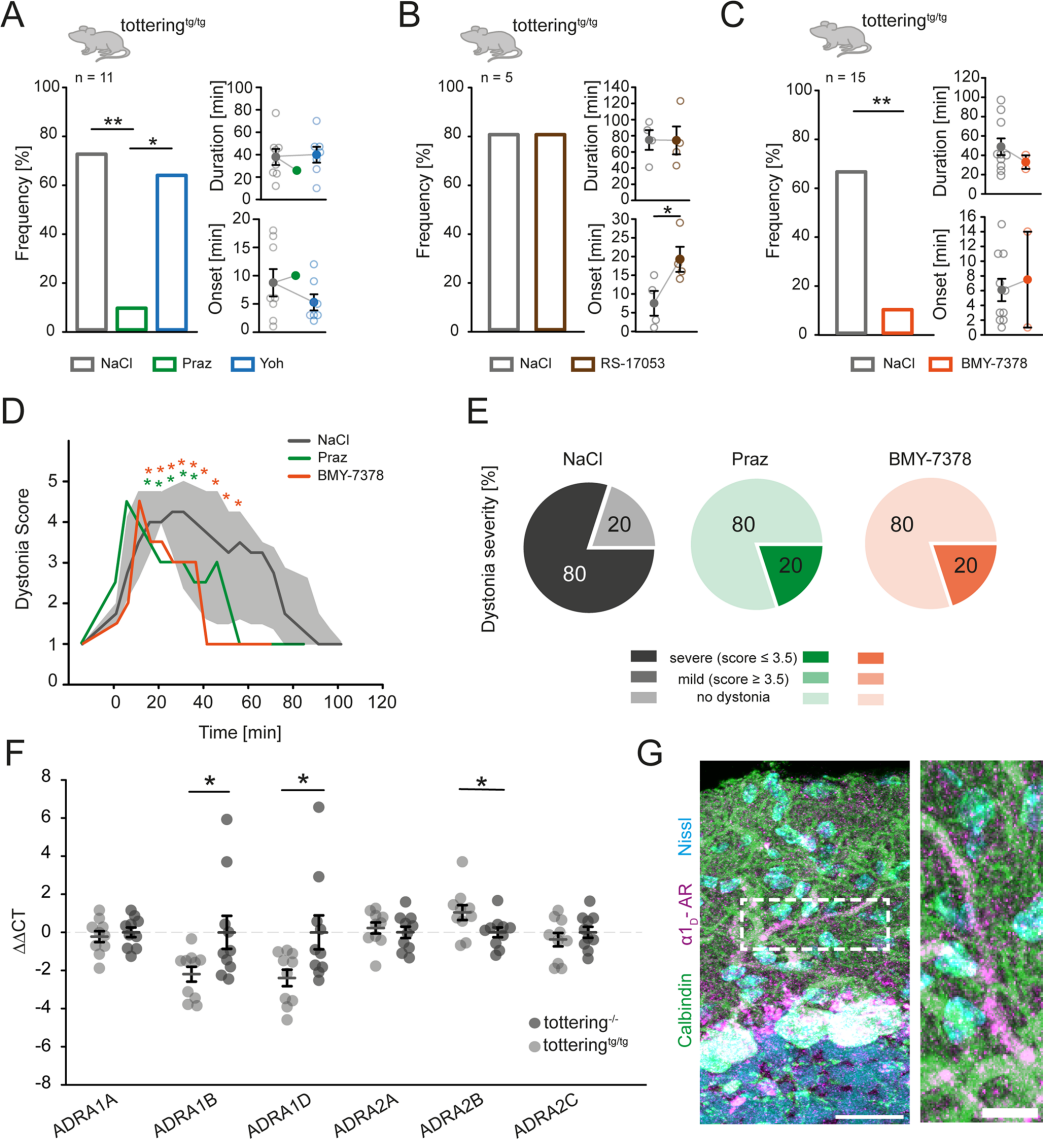
α1 adrenergic receptor antagonists prevent stress-induced dystonia in tottering^tg/tg^ mice. Efficiency of adrenoreceptor antagonists in blocking stress-induced dystonic attacks in homozygous tottering mice were evaluated using the cage change stress paradigm. (**A**) 72% of vehicle (NaCl) injected tottering^tg/tg^ mice (*n* = 11) respond with paroxysmal dystonia to the stress-test starting after 8.7 ± 2.4 min and 37.8 ± 7.1 min duration. Injection of 10 mg/kg prazosin hydrochloride (Praz), a general α1 adrenergic receptor antagonist 30 min prior to the test, reduced the frequency of mice displaying dystonia significantly to 9% (*p* = 0.003). Only one attack was observed after 10 min with a duration of 27 min. Injections of 20 mg/kg yohimbine hydrochloride (Yoh), an α2 adrenoreceptor antagonist, did neither reduce frequency of stress-induced dystonia (64%, *p* = 0.684), nor influence onset (5.2 ± 1.4 min, *p* = 0.256) or duration (39.9 ± 7 min, *p* = 0.847) of attacks. (**B**) The α1_A_-AR antagonist RS-17053 did neither alleviate stress-induced dystonia (80% vehicle, 80% RS, *p* = 1), nor influenced dystonia duration (74.75 ± 12.195 NaCl, 74.25 ± 17.24 RS, *p* = 0.982), but delayed onset of attacks from 7.5 ± 3.304 min to 19.25 ± 3.351 min (*p* = 0.047) in tottering^tg/tg^ mice. (**C**) Injections of 10 mg/kg BMY-7378 dihydrochloride, a subtype specific α1_D_-AR antagonist significantly reduced the frequency of dystonia to 13% compared to vehicle injected tottering^tg/tg^ mice (66%, *p* = 0.004, *n* = 15), while duration (48.8 ± 8.6 min NaCl, 33 ± 7 min BMY, *p* = 0.451) and onset (6.1 ± 1.5 min NaCl, 7.5 ± 6.5 min BMY, *p* = 0.745) were not affected. (**D**) Both Praz and BMY-7378 alleviated the severity of stress-induced dystonic attacks when they occurred compared to NaCl injected mice starting 15 min after exposure to stress. Data presents the severity of dystonic episodes from mice undergoing a dystonic episode after either i.p. injection of Praz (1 out of 11 mice injected) or BMY-7378 (2 out of 15 mice injected). (**E**) 80% of mice displayed severe motor symptoms undergoing stress-induced dystonia (score ≤ 3.5) after NaCl application and cage change stress, while 20% did not display an attack (score ≥ 3.5). Both Praz and BMY increased the number of mice showing no dystonia to 80%, while 20% still displayed severe motor impairments. (**F**) Analysis of the AR subtypes mRNA levels in the cerebella of both control and tottering^tg/tg^ mice via qPCR reveals that α1_B_- and α1_D_-AR mRNA are significantly upregulated in tottering^tg/tg^ compared to control mice (*p* = 0.038; *p* = 0.045, respectively). α1_A_-ARs (*p* = 0.564), α2_A_-ARs (*p* = 0.592) and α2_C_-ARs (*p* = 0.410) were not differentially expressed in tottering^tg/tg^ compared to tottering^−/−^ control mice. However, α2_B_-AR mRNA was downregulated (*p* = 0.04). (**G**) IHC staining against the α1_D_-AR (magenta) showed expression on the PC (green) dendritic tree. Scale bars: 25 μm (left) and 15 μm (right). See table S9 for data and *p*-values. Data are presented as mean ± SEM or median ± 75%/25% quartiles (**D**). Statistical significance was evaluated with student’s t-test or Mann-Whitney Rank Sum test or Two-Way Repeated Measure ANOVA. (*p ≤ 0.05, **p ≤ 0.01, ***p ≤ 0.001)

Noradrenergic blockade of α1-ARs was shown to prevent stress-induced attacks in the EA2 mouse model tottering^tg/tg^ [20, 21]. However, it is not clear whether and to what extent the specific α1-AR subtypes contribute to the formation of stress-induced dystonia. To investigate this, we initially verified the reported predominant contribution of α1-ARs, but not α2-ARs in stress-induced motor dysfunction [20] (Fig. 1A). Indeed, injection of the α1-AR antagonist prazosin hydrochloride (Praz) 30 min prior to cage change stress significantly alleviated the frequency of stress-induced motor dysfunction in tottering^tg/tg^ mice but did not affect the onset or duration in animals experiencing a dystonic episode. Strikingly, permanent ataxia reported in both EA2 patients [5, 23] and tottering^tg/tg^ mice [10] was not improved in presence of Praz (Table S1). Furthermore, injection of the α2-AR antagonist yohimbine hydrochloride (Yoh) did not affect the frequency or duration of stress-induced attacks. However, it did show a trend in delaying attack onset (Fig. 1A), thereby confirming the postulated predominant role of α1-ARs in stress-induced dystonia.

Next, we injected tottering^tg/tg^ mice with AR specific antagonist to further dissect the involvement of the three α1-AR subtypes in stress-induced paroxysmal dystonia. Mice were injected with either RS-17053, an α1_A_-AR specific antagonist (Fig. 1B), or BMY-7378, an α1_D_-AR specific antagonist (Fig. 1C) [24] 30 min prior to a cage change. Inhibition of α1_A_-ARs had no impact on the probability of stress-induced dystonia or duration of attacks but significantly delayed the onset of attacks in response to stress (Fig. 1B). In contrast, inhibition of α1_D_-ARs effectively prevented stress-induced dystonic episodes in tottering^tg/tg^ mice with tendencies to reduce attack duration (Fig. 1C). In addition, BMY-7378 injection partially improved ataxia, but not muscle strength, in these mice (Table S2). In tottering^tg/tg^ mice, episodes of dystonic attacks follow a characteristic uniform progression starting with extension of their hind limbs, followed by an overarched back, twitching forelimbs and a stiff neck before reaching the head, which results in prolonged immobility of the mouse [9, 10]. This uniformity allowed us to score the severity of dystonic attacks using a modified scale [25] (Fig. 1D, E). Those few dystonic attacks that still proceeded in presence of Praz or BMY-7378, reached identical severities to attacks caused by NaCl injection in the first 20 min, but showed a significant milder course and attack duration (Fig. 1D, E). These data strongly suggest a predominant role for α1_D_-ARs in the formation of stress-induced dystonia.

To further strengthen this hypothesis, we analyzed cerebellar AR expression. A study performed in the late 80 s found slight overall α_1_-AR upregulation in the cerebellum of tottering^tg/tg^ mice via receptor audiography with [^3^H]prazosin [26]. However, the individual α1-AR subtypes were not further characterized. Additionally, AR localisation on different cell types within the cerebellum yielded different results, depending on the method used and species analysed [27–29]. For α1_D_-ARs, a study in humans found expression restricted to PCs and DCN [29]. However, adrenergic receptor expression may be different in EA2 patients and tottering^tg/tg^ mice, which could partially explain their increased susceptibility to stressors. To identify the different AR subtypes in the cerebellum, we performed qPCR of cerebellar ARs (Fig. 1F) and conducted supporting IHC localization staining (Figure S1). mRNA analysis revealed altered AR expression in tottering^tg/tg^ compared to control mice. Strikingly, cerebellar α1_D_-AR mRNA as well as α1_B_-AR mRNA were significantly upregulated in tottering^tg/tg^ mice. α2_B_-ARs displayed a reduced expression in tottering^tgtg^ compared to control littermates (see Table S9 for data and *p*-values). Unfortunately, little is known about AR expression in the murine cerebellum, thus our observation currently eludes our understanding, but may be a consequence of the NE hyperinnervation, which was correlated to upregulation of α1-AR expression [26]. Staining against the α1_D_-AR subtype displays localization on the PC dendritic tree (Fig. 1G), possibly postsynaptic, which was not observed for the other subtypes (Figure S1). Taken together, our data suggest that α1_D_-AR may primarily be located at the PC dendritic tree and may play a role in mediating stress-induced dystonia in tottering^tg/tg^ mice.

### In vivo microinjection of the α1_D_-adrenoreceptor antagonist BMY-7378 rescues norepinephrine-induced inhibition of cerebellar Purkinje cells

**Fig. 2:**
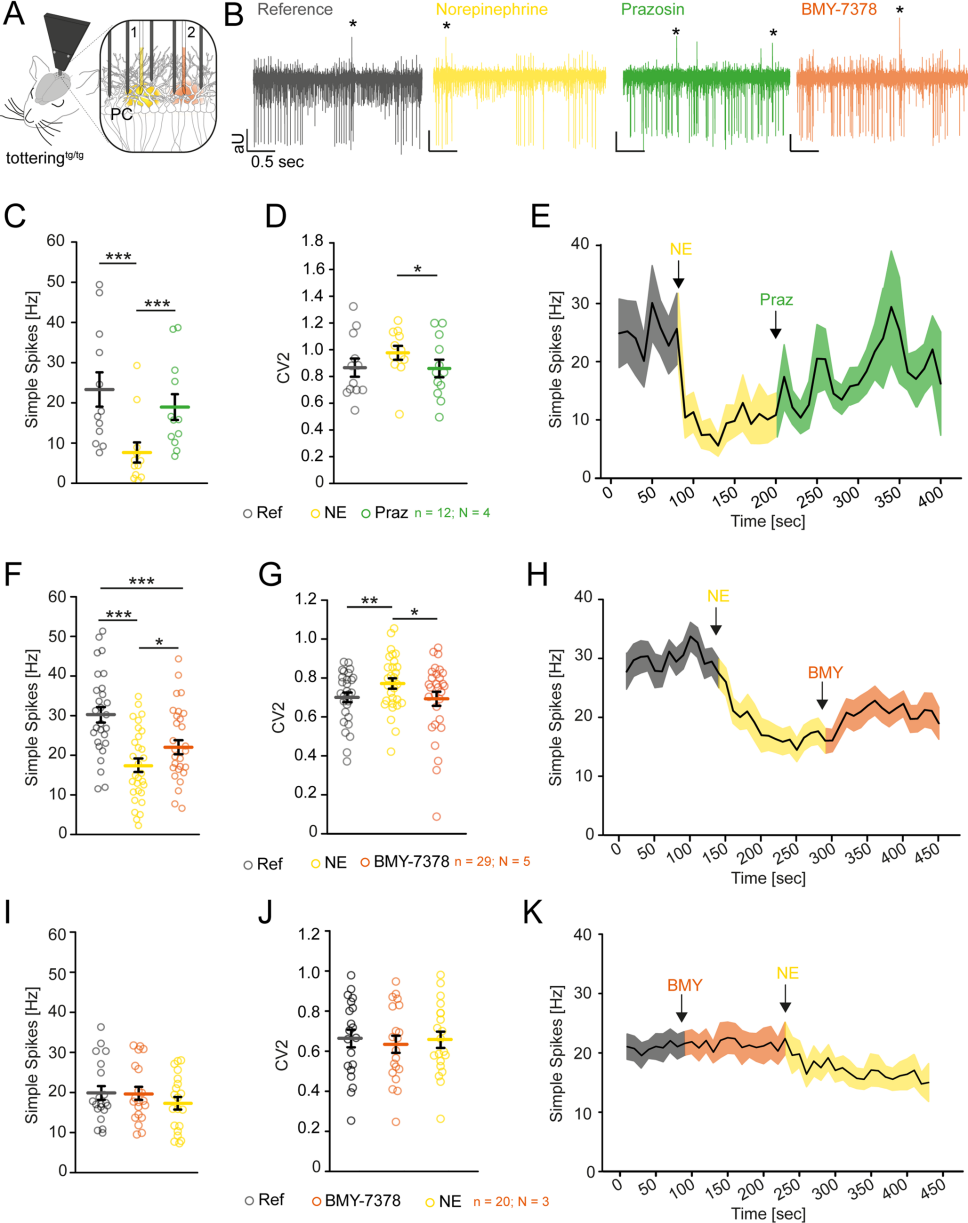
The general α1AR antagonist Prazosin hydrochloride and subtype-specific α1_D_ AR antagonist BMY-7378 restore Purkinje cell firing in vivo in tottering^tg/tg^ mice. (**A**) Schematic of the in vivo recording setup. NE [1] and AR blockers [2] were pressure injected via microinjection pipettes and PC activity was simultaneously recorded via 5 electrodes. (**B**) Example traces of spontaneous PC activity (reference, grey), after application of NE (yellow), Praz (green) and BMY-7378 (orange). (**C**) Praz application recovers PC SS firing after NE-mediated inhibition (7.65 ± 2.51 vs. 18.942 ± 3.209, *p* ≤ 0.001, *n* = 12, *N* = 4) and restores the regularity indicated by the CV2 value (0.977 ± 0.0524 vs. 0.860 ± 0.0657, *p* = 0.034) (**D**). (**E**) Mean trace of PC firing after NE and Praz injection. (**F**) The α1_D_-AR subtype specific blocker BMY-7378 alleviates NE-mediated inhibition of PC SS firing (17.486 ± 1.711 vs. 22.031 ± 1.77, *p* = 0.013, *n* = 29, *N* = 5). (**G**) NE application significantly increased PC irregularity (0.772 ± 0.0268, *p* = 0.002), which was recovered to reference levels after BMY application (0.693 ± 0.0362, *p* = 0.015). (**H**) Mean trace of PC firing after NE and BMY-7378 application. (**I-K**) Application of BMY-7378 before pressure injection of NE effectively prevented the NE-induced inhibition of PC SS firing (*n* = 20, *N* = 3) (**I**) and promoted stable firing regularities (**J**). (**K**) Mean traces of PC firing when BMY-7378 is applied before NE. See table S9 for data and *p*-values. Data are presented as mean ± SEM. Statistical significance was evaluated by paired t-test. (*p ≤ 0.05, **p ≤ 0.01, ***p ≤ 0.001)

Release of NE was previously shown to inhibit PC firing [30] and induce erratic PC firing in tottering^tg/tg^ mice [21]. The loss of timely precise PC pacemaking is suggested to be the pivotal factor in cerebellar dysfunction [31]. We therefore explored the direct effects of α1_D_-AR inhibition on PC activity by electrophysiological in vivo recordings (Fig. 2). Initial extracellular in vivo recordings of PCs in both control tottering^−/−^ and tottering^tg/tg^ mice confirmed that PC firing rates are not decreased in tottering^tg/tg^ mice, but display increased irregularity in SS firing compared to control mice [32] (Figure S3, Table S10). The frequency of complex spikes (CS) was significantly reduced compared to control tottering^−/−^ mice, suggesting altered climbing fiber (CF) synaptic innervation in tottering^tg/tg^ mice (Figure S3C, Table S10). Since CF inputs trigger synaptic plasticity at both the PC dendrites and independently generate a strong distal output signal in the PC axon [33], this decrease may hint to PC coding deficits in tottering^tg/tg^ mice. Next, we hindered PC firing of tottering^tg/tg^ mice by pressure injection of 20 µM NE, a dose previously reported to activate α1-ARs in PCs [34] (Figure S4, Table S10). We observed a 53% decrease of PC SS firing (Figure S4C), increased irregularity of PC SS firing (Figure S4E, Table S10) and a 53% reduction of CS which did not recover during recordings (Figure S4F, J). Therefore, our in vivo NE application resembles PC irregularity observed during and identified as the cause for caffeine [35] and stress-induced motor attacks [21].

We next dissected the effects of α1- and α2-AR blockers on NE-mediated inhibition of PCs (Fig. 2, Figure S5−6) [17, 30, 36]. The general α1-AR blocker Praz reliably recovered PC SS firing to 81% of reference levels after NE-mediated inhibition (Fig. 2C) and successfully restored PC regularity as indicated by a restored CV2 (Fig. 2D). Since the α1_D_-AR specific antagonist BMY-7378 reduced the frequency and severity of dystonia in tottering^tg/tg^ mice, we investigated the putative protective effects of BMY-7378 on PC pacemaking (Fig. 2F-H). The dose of BMY was established beforehand in control animals (Table S3). Although less effective than Praz, BMY-7378 injection after NE-mediated inhibition still recovered PC SS firing by 26% (Fig. 2F) and successfully recovered PC regularity to reference levels (Fig. 2G) without altering PC CS activity (Figure S5). Strikingly, when BMY-7378 was applied 60 s before NE injection, we found protective effects of the α1_D_-AR blocker on PC SS firing (Fig. 2I-K). The NE-induced inhibition of PC firing was reduced to 12% (Fig. 2I) and the regularities of recorded cells were also preserved (Fig. 2J). The CV values for SS and CS for the different conditions are presented in Table S4. These data suggest that NE decreases PC regularity predominantly through α1_D_-ARs.

To minimize the chance of measuring false positive effects from the pressure injection, we performed control recordings using the α2-AR antagonist Yoh (Figure S6). Following NE-induced PC SS inhibition, subsequent Yoh injection neither recovered, nor further decreased PC SS and CS firing in tottering^tg/tg^ mice (Figure S6B-G), but did increase PC irregularity (Figure S6C). These results are in accordance with our behavior data (Fig. 1A).

Taken together, our recording data suggest that NE directly and indirectly alters both the SS firing and regularity of PCs in tottering^tg/tg^ mice via α1_D_-ARs. Major influences by β-ARs on PC firing have been ruled out previously [21]. Since NE has been shown to have bimodulatory effects on PC firing [34] through activation of α1-ARs on molecular layer interneurons (MLIs) resulting in GABAergic potentiation [16, 30], we did not rule out secondary effects of BMY-7378 on other cerebellar neurons that help to recover PC activity.

### Chemogenetic inhibition of molecular layer interneurons does not alleviate stress-induced dystonia

Past studies showed that NE enhances presynaptic GABA release onto PCs by binding to postsynaptic α1-ARs on MLIs [37] and that electrical stimulation of MLIs mimics NE-like inhibitory effects on PCs [38], suggesting a MLI contribution to dystonia formation. Thus, we cannot rule out secondary effects of BMY-7378 on PC SS recovery by blocking α1_D_-ARs on cerebellar MLIs, thereby preventing the reported GABAergic potentiation.

**Fig. 3:**
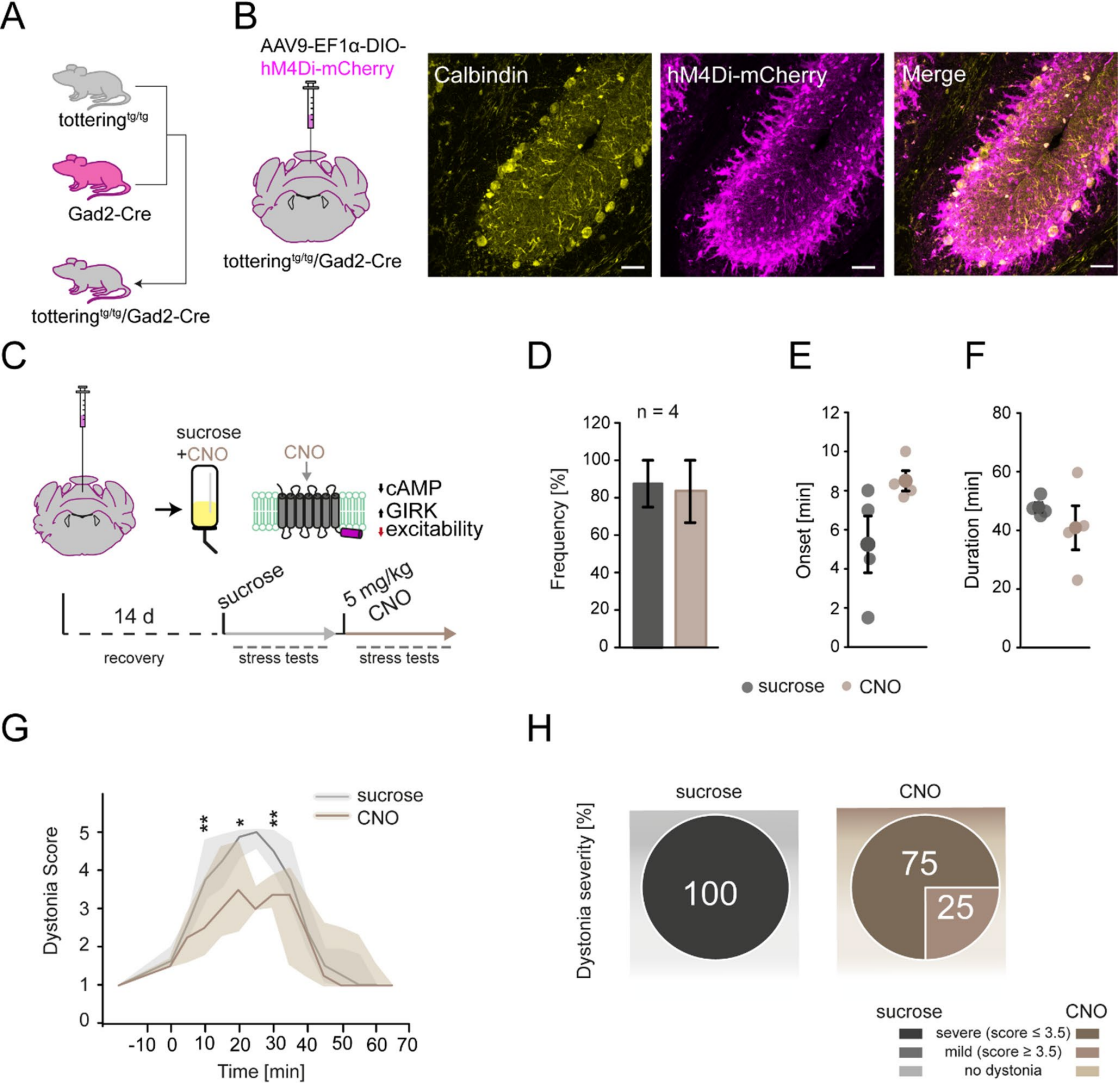
Chemogenetic silencing of cerebellar molecular interneurons does not alleviate stress-induced paroxysmal dystonia in tottering^tg/tg^/Gad2-Cre mice. (**A**) tottering^tg/tg^ were bred with Gad2-Cre mice to obtain tottering^tg/tg^/Gad2-Cre mice to allow for selective expression of CRE in cerebellar molecular interneuron (MLIs). (**B**) AAV9-EF1α-DIO-hM4Di-mCherry was injected into the vermis of tottering^tg/tg^/Gad2-Cre mice (*n* = 4). Example images showing expression of hM4Di-mCh in cerebellar MLIs (magenta), but not PCs (yellow). Scale bar: 50 μm. (**C**) Scheme of the testing paradigm. After surgery, mice recovered for 14 days, then received 2% sucrose in drinking water for 5 days and were tested in the cage change stress test. Afterwards, mice received 5 mg/kg CNO in 2% sucrose to activate the inhibitory DREADD receptor and were tested in the cage change test again. (**D**) Silencing of vermal hM4Di-mch expressing MLIs via CNO did not alleviate stress-induced dystonia compared to sucrose consumption alone (*p* = 0.537), but showed a trend to delay its onset (5.25 ± 1.451 vs. 8.5 ± 0.518, *p* = 0.079) (**E**). The dystonia duration was not altered (47.875 ± 1.625 vs. 40.875 ± 7.503, *p* = 0.397) (**F**). (**G**) There were significant differences for the dystonia score in DIO-hM4Di-mCherry injected mice drinking sucrose (grey line) compared to sucrose + CNO (brown line) consumption (*p* = 0.029). Thus, the dystonia score was slightly lower in mice consuming CNO. (**H**) The comparison of the dystonia severity showed no difference for the consumption of sucrose or sucrose + CNO. Data are presented as mean ± SEM, except (**G**) is presented as median + 25%/75% percentiles. See table S9 for data and *p*-values. Statistical significance was evaluated with student’s t-test or Two-Way repeated measure ANOVA. (*p ≤ 0.05, **p ≤ 0.01, ***p ≤ 0.001)

To explore this hypothesis, we used a chemogenetic approach using the inhibitory DREADD receptor hM4Di to chronically inhibit MLI activity and explore their role on stress-induced dystonia formation. DREADDs allow for a non-invasive manipulation of the neuronal network via administration of an exogenous drug, clozapine-N-oxide (CNO), which can be administrated either through i.p. injection or drinking water [39, 40]. We decided for the oral *ad libitum* consumption of CNO since a restraint for injection provokes stress-induced dystonic episodes in tottering^tg/tg^ mice [9]. We bred tottering^tg/tg^ mice with Gad2-Cre mice (Fig. 3A) to selectively express Cre in cerebellar MLIs. The injection of AAV9-EF1α-DIO-hM4Di-mCherry in the vermis resulted in selective expression of the hM4Di receptor in MLIs (magenta) but not PCs (yellow) (Fig. 3B). After recovery, mice were initially administrated 2% sucrose in their drinking water for a week and underwent the cage change stress test to explore the effects of sucrose consumption alone on stress-induced dystonia, before adding 5 mg/kg CNO to the solution for the subsequent week (Fig. 3C). We found that after oral administration of CNO for five days, no alleviation of stress-induced dystonia could be observed during the cage change test compared to sucrose consumption alone (Fig. 3D). We did, however, observe a trend in delaying the dystonia onset after CNO consumption compared to sucrose alone (8.5 ± 0.518 vs. 5.25 ± 1.451, *p* = 0.079), while the dystonia duration was not altered (Fig. 3E-F). We did observe a reduction in dystonia severity at limited time points after consumption of CNO compared to sucrose consumption alone (Fig. 3G-H), Therefore, we conclude that the contribution of MLIs to dystonia formation is negligible, since chemogenetic inhibition of MLIs and expected reduction of GABA release onto PCs was unable to prevent stress-induced dystonia, but modulated dystonia severity. Thus our experiment weakens the hypothesis of a MLI contribution to dystonia in tottering^tg/tg^ mice, thereby strengthening the predominant role of PCs in dystonia [31]. Since CNO is 20x less potent at inhibitory compared to excitatory DREADD receptors [41], the ultimate role of the cerebellar network contribution to PC SS firing and dystonia formation remains to be determined.

### Chronic delivery of BMY-7378 in the cerebellum of tottering^tg/tg^ mice abolishes stress-induced dystonia

**Fig. 4:**
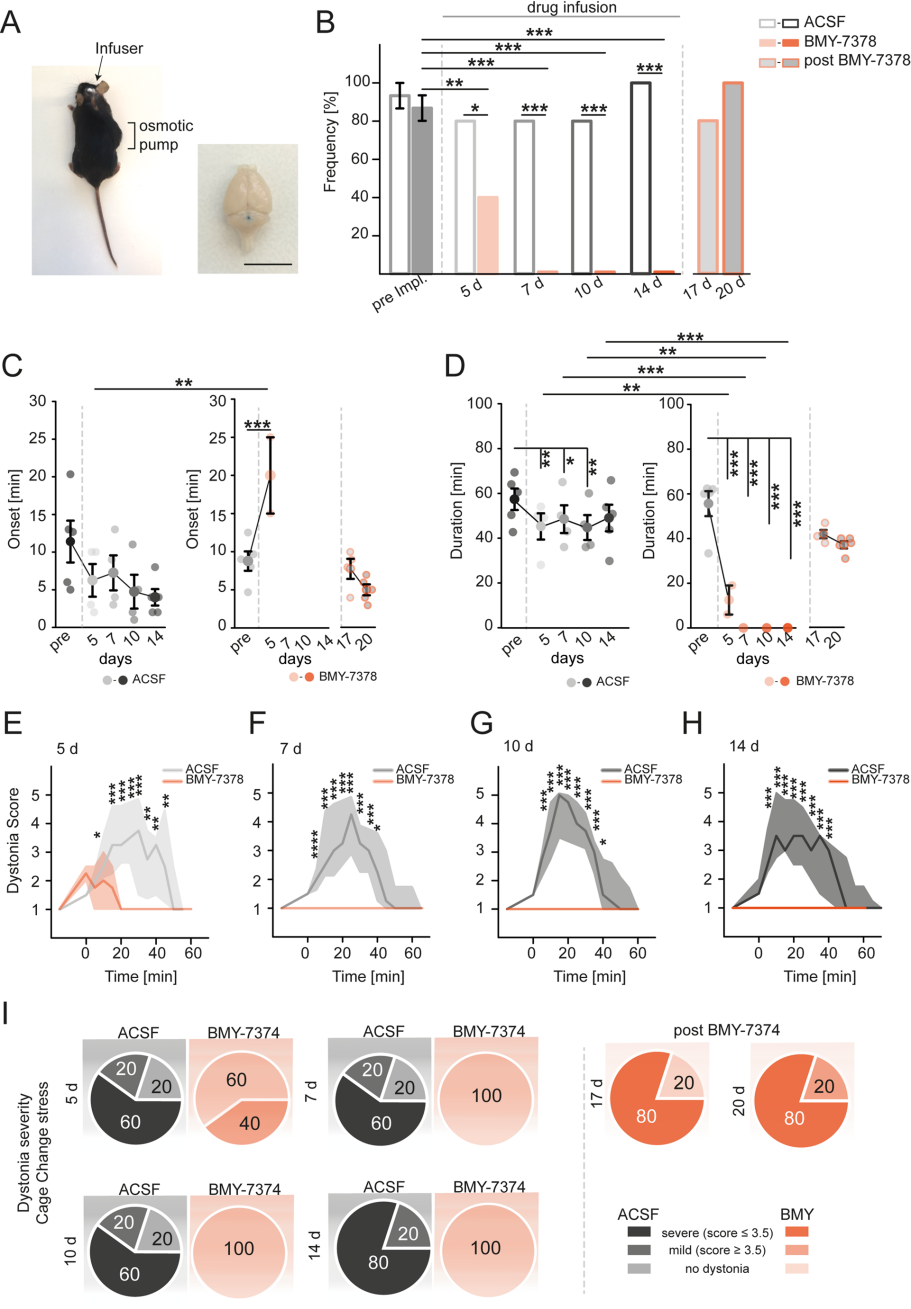
Intracerebellar chronic infusion of BMY-7378 alleviates stress-induced paroxysmal dystonia in tottering^tg/tg^ mice. (**A**) The osmotic pump was implanted subcutaneously at the animals’ right side, a tubing connected the pump to the infuser, allowing chronic intracerebellar infusion of drugs. Canula placement was verified by post-mortem injection of Evans blue stain. (**B**) Intracerebellar chronic delivery of the α1_D_-AR antagonist BMY-7374 effectively prevented stress-induced dystonic attacks starting 5 d after surgery by 50% compared to ACSF infused mice and were completely abolished after 7 d (each *n* = 5). Dystonic attacks returned after 17 d, due to drug depletion after 14 d, eliminating cerebellar damage as a contributing factor to preventing dystonia. (**C**) The onset of dystonia in ACSF infused tottering^tg/tg^ mice showed a trend in reduction starting 11.4 ± 2.775 min pre-implantation and 4.0 ± 1.095 min after 14 d of infusion (*p* = 0.435). BMY infused mice onset was delayed from 8.766 ± 1.268 min to 20.0 ± 5.0 min after 5 d (*p* ≤ 0.001) and recovered to pre-implantation levels after infusion of the drug stopped (7.75 ± 1.315 min, 17 d). (**D**) Dystonia duration was significantly reduced from 55.632 ± 5.593 min to 12.5 ± 6.5 min after 5 d (*p* ≤ 0.001) or completely abolished in α1_D_-AR blocker infused mice (*p* ≤ 0.001), though dystonia duration recovered to pre-implantation levels after delivery of BMY (42.0 ± 1.871 min, 17 d). ACSF infused mice displayed reduced dystonia duration at certain time points tested (57.365 ± 4.809 min pre, 45.25 ± 5.851 min 5 d (*p* = 0.003), 48.5 ± 6.198 min 7 d (*p* = 0.012), 44.75 ± 5.558 min 10 d (*p* = 0.003)), though there was no overall reducing trend observed. (**E-H**) Comparison of dystonia severity revealed an alleviation of severity already after 5 d of BMY infusion, while ACSF infusion did not alleviate severity at any given time. (**I**) During chronic infusion of ACSF, 60–80% of tottering^tg/tg^ mice displayed severe dystonic attacks, while none of BMY infused animals did. Severe paroxysmal dystonic attacks were observed after BMY infusion stopped (80%). See table S9 for data and *p*-values. Data are presented as mean ± SEM or median ± 75%/25% quartiles (**E**-**H**). Statistical significance was evaluated by Two-Way repeated measure ANOVA (*p ≤ 0.05, **p ≤ 0.01, ***p ≤ 0.001)

Based on our data, the α1_D_-AR expressed in the cerebellum may be a promising target to alleviate stress-induced dystonia. Our data and several past studies only provide a correlative link of episodic motor dysfunction to cerebellar dysfunction or disruptions of PC firing [22, 35]^42^– [44]. Since the cerebella of tottering^tg/tg^ mice are LC-NE hyperinnervated [19] and optogenetic activation of the LC in tottering^tg/tg^ mice is sufficient to provoke episodes of motor dysfunction [21], blocking specifically cerebellar α1_D_-ARs should prevent or at least decrease the probability of stress-induced dystonia. To test this hypothesis, we chronically infused BMY-7378 or vehicle alone directly into the cerebellum of tottering^tg/tg^ mice using osmotic pumps [31, 35] (Fig. 4). Five days after surgery, BMY-7378 infused mice manifested fewer stress-induced dystonic attacks coupled with a delayed onset and decreased duration compared to vehicle infused mice (Fig. 4B-D). Strikingly, dystonic attacks were completely abolished after 7 days of drug infusion lasting another 7 days until depletion of the pumps, while vehicle infused mice permanently displayed stress-induced dystonia (Fig. 4B). After depletion of the pump the frequency, onset and duration of dystonic episodes returned to pre-surgery levels (Fig. 4B-D), thereby eliminating cerebellar damage as a contributing factor to preventing episodic dystonic attacks [45]. Vehicle infused mice displayed an accelerated responsiveness to the cage change stress as indicated by reduced onset of attacks (Fig. 4C), while their dystonia duration remained unaltered (Fig. 4D). Although BMY-7378 infused mice still displayed dystonia 5 days post infusion, scoring of dystonia severity revealed significantly milder attacks (Fig. 4E, I), while severity of episodic dystonic attacks of vehicle infused tottering^tg/tg^ mice were not diminished (Fig. 4E-I). Dystonia severity increased again after pump depletion in BMY-7378 infused tottering^tg/tg^ mice (Fig. 4H). Collectively, these data verify that cerebellar α1_D_-ARs are the predominant AR subtype involved in the formation of stress-induced dystonia in tottering^tg/tg^ mice, providing compelling evidence that antagonizing α1_D_-ARs decrease the frequency and severity of dystonic episodes.

### Cerebellar shRNA-mediated knock-down of α1_D_ adrenergic receptors prevents stress-induced dystonia

**Fig. 5:**
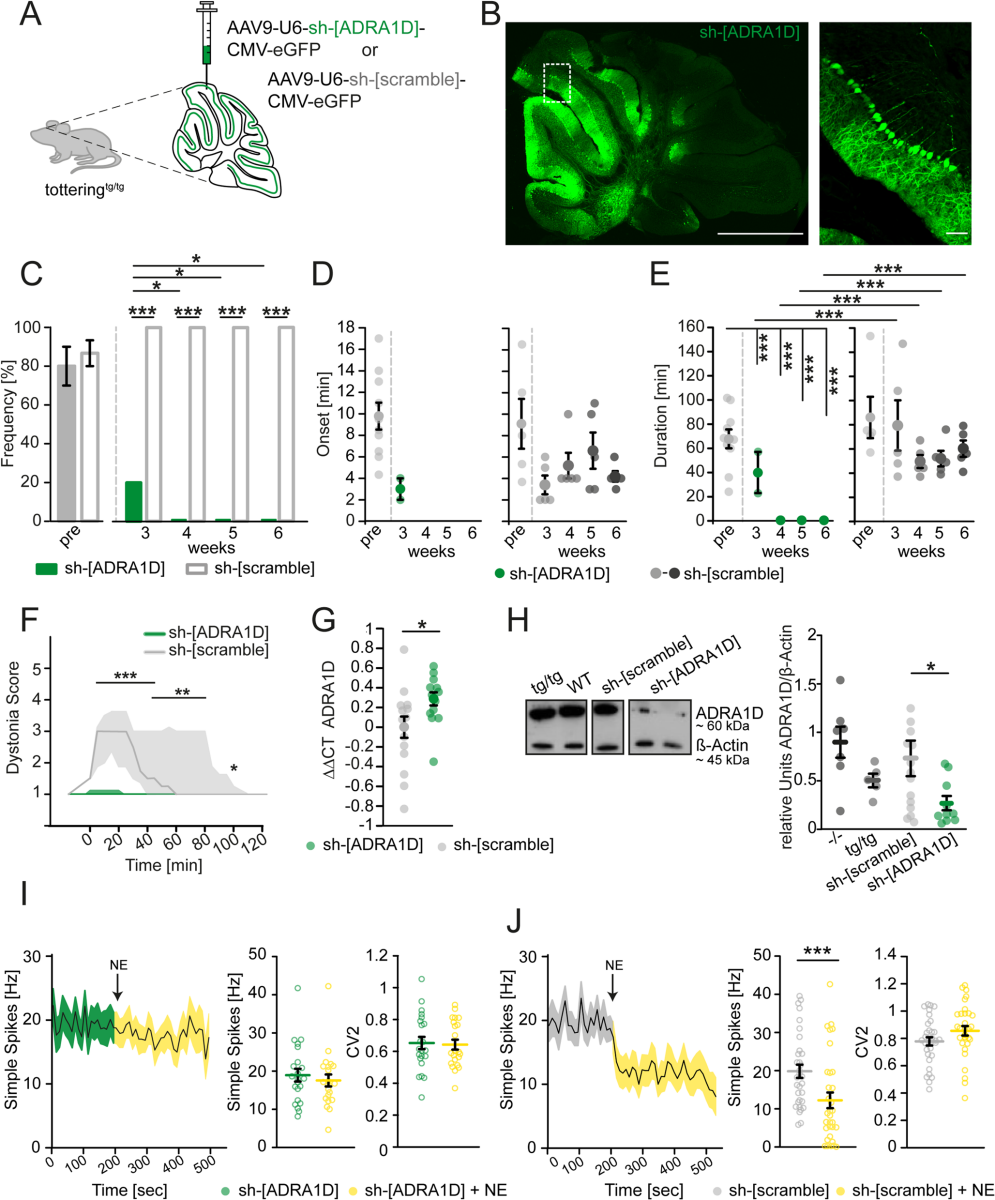
ShRNA-mediated knock-down of cerebellar α1_D_ adrenergic receptors prevents stress-induced dystonia. (**A**) Targeting AAV9-U6-sh-[ADRA1D]-CMV-eGFP or non-targeting AAV9-U6-sh-[scramble]-CMV-eGFP were injected into the cerebella of tottering^tg/tg^ mice (*n* = 10; *n* = 5, respectively). (**B**) Example images verifying robust expression of sh-[ADRA1D] virus (green) in the anterior cerebellum after 6 weeks of expression. Scale bar 1 mm. Close-up images showing individual PCs expressing the sh-[ADRA1D] virus, Scale bar: 50 μm. (**C**) After 3 weeks of expression, sh-[ADRA1D] (green) injected mice significantly displayed fewer stress-induced motor attacks than sh-[scramble] control mice (grey 20% vs. 100%, *p* ≤ 0.001). After 4 weeks, stress-induced dystonia was abolished completely, while sh-[scramble] continued to experience attacks (100%). (**D**) Onset of stress-induced dystonia was not statistically analyzed with the Two-Way RM ANOVA since sh-[ADRA1D] injected mice did not suffer from dystonia. Onset of attacks was not altered in control mice (*p* = 0.347, One-Way RM ANOVA). (**E**) Compared to pre-injection dystonia duration (67.816 ± 7.782 min), mice with α1_D_-AR knock-down showed significant decrease of attack duration to 40 ± 17 min. No attacks were present after 4 weeks, while attack duration in sh-[scramble] injected mice was not altered. **(F)** sh-[ADRA1D] expressing mice experiencing attacks at 3 weeks post injection had significantly milder dystonia compared to sh-[scramble] injected mice. (**G**) Reduced expression of α1_D_-AR mRNA was found in sh-[ADRA1D] injected mice (green, *n* = 14) compared to control mice (grey, *n* = 15) (0.287 ± 0.0665 vs. 0 ± 0.0108; *p* = 0.035) by qPCR. (**H**) Example western blot depicting protein levels of ADRA1D and β-actin in WT (*n* = 7), tottering^tg/tg^ (*n* = 5), sh-[scramble] (*n* = 14) and sh-[ADRA1D] (*n* = 10) injected mice. Quantitative analysis revealed a significant knock-down of the α1_D_-AR protein (~ 60 kDa) in sh-[ADRA1D] injected mice (*n* = 10, 0.277 ± 0.0734) compared to the sh-[scramble] injected control group (*n* = 14, 0.738 ± 0.183) (*p* = 0.038). (**I**) In vivo PC recordings of sh-[ADRA1D] expressing tottering^tg/tg^ mice showed a diminished reduction in their SS firing (7.4%) after pressure injection of NE and maintained their firing regularity as indicated by the CV2. (**J**) Purkinje cells expressing the control sh-[scramble] showed a significant reduction of their SS firing by 38% (pre = 19.837 ± 1.726, post = 12.222 ± 2.052, *p* = 0.001). See table S9 for data and *p*-values. Data are presented as mean ± SEM or median ± 75%/25% quartiles (**F**). Statistical significance was evaluated by Two-Way repeated measure ANOVA or Student’s-t test (**G**, **H**) (*p ≤ 0.05, **p ≤ 0.01, ***p ≤ 0.001)

To specifically implicate the role of α1_D_-ARs in dystonia, we conditionally knocked-down these receptors in the anterior cerebellum using small hairpin RNAs (shRNAs). Tottering^tg/tg^ mice were stereotaxically injected with either a mixture of AAV2-CMV-eGFP-U6-mAdra1d[shRNA#1–3] (sh-[ADRA1D]) targeting three different α1_D_ mRNA sequences or non-targeting AAV2-CMV-eGFP-U6-scramble (sh-[scramble]), resulting in robust eGFP expression in cerebellar PCs (Fig. 5A-B). eGFP expression was found in both Zebrin II^+^ as well as Zebrin II^−^ PCs in different lobules (Figure S7). Starting three weeks post injection, both sh-[ADRA1D] and sh-[scramble] injected mice were tested in the cage change stress paradigm to determine their susceptibility to stress-induced dystonia (Fig. 5C). sh-[ADRA1D] injected mice showed a significant reduction of stress-induced dystonia after three weeks of expression compared to sh-[scramble] injected control tottering^tg/tg^ mice, but also a faster onset (Fig. 5D) and a reduction in dystonia duration (Fig. 5E). Since only two out of ten sh-[ADRA1D] injected mice displayed stress-induced dystonia, no statistical analysis could be performed. After four weeks, stress-induced motor attacks were prevented completely in sh-[ADRA1D] injected mice (Movie S1), while sh-[scramble] injected tottering^tg/tg^ mice continued to display stress-induced dystonia without alterations in onset or duration of motor attacks (Fig. 5C-E) (Movie S2). Although 20% of sh-[ADRA1D] injected mice experienced dystonia in the third week of expression, severity of attacks was significantly reduced compared to control mice (Fig. 5F). Quantitative PCR analysis of sh-[ADRA1D] injected cerebella revealed a significant reduction of α1_D_-AR mRNA levels compared to sh-[scramble] injected tissue (Fig. 5G), confirming specificity of targeting α1_D_-AR mRNA. However, we found that the other α1-, and α2-ARs were upregulated in sh-[ADRA1D] injected compared to sh-[scramble] injected mice (Table S5), possibly due to a genetic compensation in response to gene knockout [46]. Additional western blot analysis showed significantly decreased levels of α1_D_-AR protein expression, verifying a reduction of the receptor in only sh-[ADRA1D] but not sh-[scramble] injected mice (Fig. 5H). It is noteworthy that we measured a downregulation of α1_D_-AR protein in naïve tottering^tg/tg^ compared to WT control mice (Fig. 5H), while initial α1_D_-AR mRNA levels were upregulated in tottering^tg/tg^ mice (Fig. 1F). Although we do not have a finite explanation for this discrepancy, one possible explanation may be a regulatory mechanism to protect PCs from the increased susceptibility to NE. However, this hypothesis requires detailed examination.

Despite the altered AR expression, neither the motor performance, nor the anxious behavior in the open field test was affected in sh-[ADRA1D] or sh-[scramble] injected mice (Table S6). Additionally, in vivo PC recordings of both sh-RNA injected groups showed no significant differences in PC SS and CS firing (Figure S8), but after pressure injection of NE, PC expressing sh-[ADRA1D] showed only a 7.4% reduction in their SS firing, compared to a 34% NE-induced inhibition in sh-[scramble] injected mice (Fig. 5I-J), strengthening the protective effects of α1_D_-AR blockade on PC firing in presence of NE.

Together with our drug studies both electrophysiological and behavioral in mice and shRNA results, these data suggest the involvement of cerebellar α1_D_-AR in stress-induced dystonia in tottering^tg/tg^ mice.

### In vivo calcium imaging shows a rise of intracellular calcium during early stages of stress-induced dystonia

**Fig. 6:**
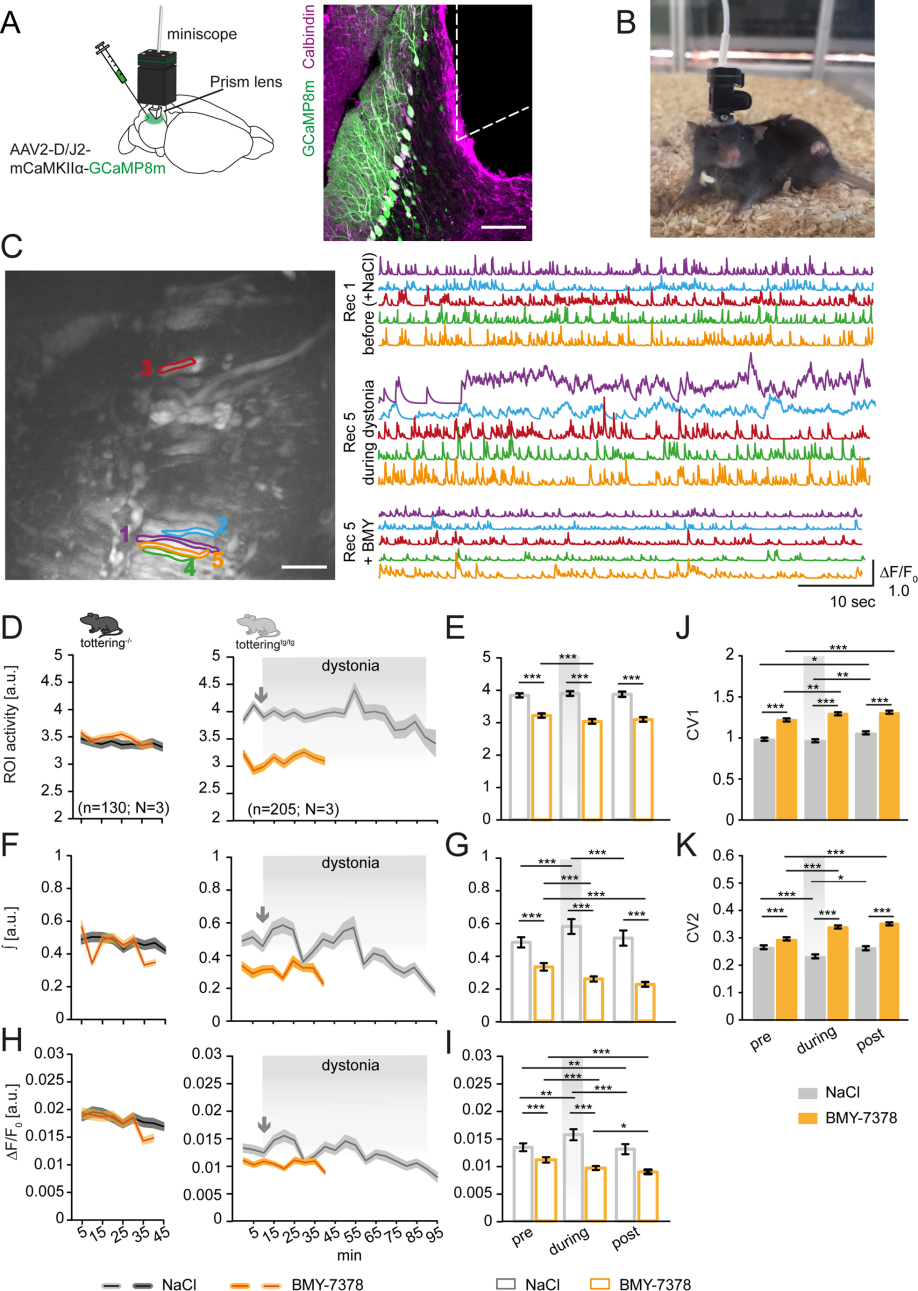
In vivo calcium imaging of Purkinje cells verifies the involvement of intracellular calcium during stress-induced dystonia in tottering^tg/tg^ mice. (**A**) Scheme of the surgery. A GCaMP8m expressing AAV was injected into the vermis, followed by prism lens implantation. Example confocal image of AAV2-D/J2-mCaMKIIα-GCaMP8m (green) showing expression only in Purkinje cells (magenta). Dashed lines indicate placement of the prism. Scale bar: 100 μm. (**B**) Image of a tottering^tg/tg^ mouse undergoing a dystonic episode while mounted to the miniscope (Inscopix). (**C**) FOV (field of view) from the Inscopix data processing software (IDPS) showing five example ROIs which we identified as PC dendritic trees and the corresponding ΔF/F_0_ (Ca^2+^) traces before and during a stress-induced dystonic episode and after BMY-7378 injection. Traces are from the same ROI and mouse, depending on the color. Over 20 ROIs were analyzed in this mouse. Scale bar: 80 μm. (**D**) Analysis of the ROI activity in tottering^−/−^ control mice display no effects of BMY-7378 i.p. injection on PC firing frequency. Tottering^tg/tg^ mice display a higher, more irregular ROI activity, which was reduced after i.p. administration of BMY-7378. (**E**) Comparison of the mean ROI activity in the first recording (0–1 min), fourth recording (15–16 min) and last recording (after dystonia ended or in case of no dystonia after 40 min) in NaCl and BMY-7378 injected tottering^tg/tg^ mice. BMY-7378 significantly decreases the mean ROI activity compared to NaCl. (**F**) Analysis of the integral (∫) as a measure of total calcium release during recordings showed that the calcium release is constant in control mice after both NaCl and BMY-7378 i.p. injection. Tottering^tg/tg^ mice displayed decreasing calcium levels, independent from the drug injected. (**G**) Comparison of the mean ∫ from the first recording (0–1 min), fourth recording (15–16 min) and last recording (after dystonia ended or in case of no dystonia after 40 min) in tottering^tg/tg^ mice showed significantly escalated amounts of calcium released at the beginning of a dystonic episode, which normalize once attacks stop. BMY-7378 injection lowers the total amount of released calcium in all recordings. (**H**) Courses of the mean fluorescence intensity (ΔF/F) in control and tottering^tg/tg^ mice demonstrated the overall relative constant fluorescence signals measured in the PC dendritic tree. In tottering^tg/tg^ mice, there is a leap of fluorescence during the early stages of dystonia, indicated by the grey arrow. (**I**) Comparison of the mean ΔF/F signals of the first recording (0–1 min), fourth recording (15–16 min) and last recording (after dystonia ended or in case of no dystonia after 40 min) in tottering^tg/tg^ mice displayed a significant increase in fluorescence as an indicator for rising calcium levels in the presence of dystonia. Signals decreased under baseline after the dystonia ceased and mice recovered from the attack. In the presence of BMY-7378 the fluorescence signals decreased, indicating lower calcium levels. Analysis of the CV1 (**J**) and CV2 (**K**) of ROIs showed significant increases of both CV1 and CV2 after BMY-7378 injection compared to NaCl injections, Light grey boxes in the back of graphs indicate the presence of dystonia throughout NaCl recordings. See table S9 for data and p-values. Data are displayed as mean ± SEM. Statistical significance was evaluated by Student’s-t test or Mann-Whitney Rank Sum test (*p ≤ 0.05, **p ≤ 0.01, ***p ≤ 0.001)

Several studies performing simultaneous single-unit electrophysiological and calcium imaging recordings in a behaving animal demonstrated that PC responses are strongly influenced by large dendritic calcium transients evoked by complex spikes [47–52]. To investigate the intrinsic mechanisms underlying the effects of BMY-7378 injection on PC firing during stressful events, we performed in vivo calcium imaging in awake, behaving tottering^tg/tg^ mice (Fig. 6). Mice were injected with a fast GCaMP8m Ca^2+^ sensor into the vermis, followed by immediate implantation of the Inscopix ProView™ Prism lens [53, 54], resulting in a robust expression of virus only in PCs (Fig. 6A). We found that tottering^tg/tg^ mice still displayed stress-induced dystonic attacks during recordings (Fig. 6B, Movie S3), despite damage to the cerebellum due to the implantation [45]. Recordings were performed in both WT (control) and tottering^tg/tg^ mice with both saline and BMY-7378 injection during a cage change test. The fluorescence signals of manual ROI of PC dendrites from a tottering^tg/tg^ mouse before, during a dystonic attack and following BMY injection were analyzed (Fig. 6C). Control mice displayed regular dendritic ROI activity, which did not change during the duration of recordings independent from the injected agent (Fig. 6D), while tottering^tg/tg^ mice showed a higher activity of ROIs as a measure for frequency (Fig. 6D, Movie S3). BMY-7378 injection significantly reduced the ROI activity in tottering^tg/tg^ mice (Fig. 6D-E, Movie S4), similar to control levels. Analysis of the integral (∫) as a measure for released calcium per recorded activity showed an overall decrease in calcium levels with ongoing recordings in tottering^tg/tg^ mice independent from the injected agent (Fig. 6F-G). We exclude decreasing sensitivity of the sensor as a reason for this, since control mice displayed almost constant levels (Fig. 6F), but rather a physiological circumstance based on the general decreased Ca^2+^ influx through P/Q-type channels in tottering^tg/tg^ mice [7]. This is also reflected in the lower ΔF/F_0_ traces of tottering^tg/tg^ compared to WT mice despite the injected drug (Fig. 6H). BMY-7378 did not recover the calcium influx but stabilized the overall calcium levels (Fig. 6H). Interestingly, we found an increase of the ROI activity right before onset of the dystonic episode, accompanied by an increase in peak height and the total amount of calcium influx as indicated by the light grey arrows (Fig. 6F-G). This likely reflects the erratic burst firing of PC reported at the beginning of dystonic episodes [21, 31]. With the ongoing of the attack, the PCs do not seem to recover well from the amounts of released calcium, as indicated by the decreasing integral (measure for total calcium released) and ΔF/F_0_ (proximity for calcium peak height). However, we cannot exclude the possibility, that the decrease of the fluorescence signal is due to bleaching of the GCaMP8m [53]. Interestingly, we found that the dendritic CV1 and CV2 was increased in presence of BMY-7378 compared to control saline injection (Fig. 6J-K). Similarly, the CV1 increased after saline injection with ongoing dystonia duration (Fig. 6J), while the CV2 pre and post dystonia was not altered (Fig. 6J).

Based on the lower calcium transients measured in tottering^tg/tg^ mice, we assume that their calcium homeostasis is impaired due to the P/Q-type channel mutation. The large calcium transients observed during calcium imaging of PCs was shown to be mediated by inferior olive-climbing fiber inputs to PCs [55], thereby reflecting altered climbing fiber inputs evoking complex spikes in PCs which is also reflected in our electrophysiological recordings (Figure S3). Our recordings of calcium activity on PC dendritic trees show rises in calcium levels at the beginning of dystonic events, however the cause for this remains elusive. Our findings strengthen the idea of impaired calcium homeostasis during stress-induced dystonia through the Gq-coupled GPCR α1_D_-AR [56].

## Discussion

Episodic ataxia type 2 is an episodic neurological disorder caused by mutations in the Ca_V_2.1 channel [44, 57, 58], where patients suffer from attacks of severe motor dysfunction elicited through emotional or physical stressors. The ataxic EA2 mouse model tottering^tg/tg^ displays ataxia and episodes of severe paroxysmal dystonia [10], which are reliably provoked by stress, the most common trigger in episodic neurological disorders [8, 9]. Stress is mediated through release of NE from the LC [59], the sole noradrenergic source for the cerebellum, where LC noradrenergic terminals almost exclusively synapse on PCs [14]. Tottering^tg/tg^ mice are LC-NE hyperinnervated [19] and noradrenergic blockade of α1-AR was shown to prevent stress-induced motor attacks [20, 21]. The cerebellum and especially PCs were shown to be predominantly responsible for the formation of motoric deficits such as dystonia in EA2, since mutations in or manipulations of the P/Q-type calcium channel trigger dystonic episodes accompanied by erratic PC firing [31, 43, 44, 58, 60]. Although other brain areas such as the motor cortex, which participate in the execution of movements, have been discussed in the formation of dystonia [61], a recent study characterized the low-frequency oscillations and calcium responses during a dystonic attack in tottering^tg/tg^ mice, but found no change in M1 neuronal activity, implicating another area of the brain responsible for the initiation or control of dystonia [62]. Here, we suggest that cerebellar α1_D_-ARs on PCs are a key player in the formation of stress-induced dystonia in tottering^tg/tg^ mice, providing a new potential target in the therapy of EA2.

The noradrenergic activation of α1_D_-ARs on PCs resulted in long-lasting inhibition of PC SS firing (Fig. 2, Figure S4) [15–18]. This inhibition of PC activity is caused by the bimodulatory effects of NE through direct excitation or indirect inhibition [34]. Activation of presynaptic β_2_-ARs on MLIs causes excitation, resulting in increased MLI firing and high potentiation of GABAergic transmission at the basket neuron to PC synapse [30, 63–66]. However, neither β-AR blockade nor chemogenetic silencing of MLIs prevented stress-induced dystonia (Fig. 3) [20, 21]. Synaptic transmission in tottering^tg/tg^ mice is mediated by slower N-type Ca^2+^ channels as opposed to the WT P/Q-type channel, which display increased sensitivity to GPCR mediated inhibition and thereby producing a 3–5 fold higher NE-driven synaptic inhibition through GABA_B_-R and α2-AR [67]. Additionally, NE binding to presynaptic α1-ARs on MLIs increases their firing [30, 68], and simultaneous binding to postsynaptic α1-ARs enhances MLI presynaptic inhibitory transmitter release onto PCs [37], supporting the theory that postsynaptic α1-ARs, specifically α1_D_-ARs on PCs, are the main driver of stress-induced dystonia. In agreement with this theory, we show that pharmacological blockade of α1_D_-ARs in the cerebellum by BMY-7378 [24] diminished, but not abolished the NE-induced SS depression of PCs, thereby suggesting that NE enhances the GABA-mediated inhibition of PC SS through postsynaptic α1_D_-ARs. In addition to altered NE innervation [69], we show that cerebellar α1_D_-ARs mRNA levels are increased in tottering^tg/tg^ (Fig. 1F). Moreover, immunohistochemical staining indicates expression mainly on PCs, likely at postsynapses [21, 70] (Fig. 1G). In accordance with our findings, a study in humans also found α1_D_-AR mRNA exclusively in PCs [29]. Our findings contribute significantly to the understanding of why PCs of tottering^tg/tg^ mice display increased susceptibility to NE-mediated inhibition (Fig. 2). Consequently, pharmacological blockade of cerebellar α1_D_-AR through BMY-7378 rescued PC SS firing (Fig. 2J-L) and prevented stress-induced dystonia (Figs. 1C and 4). However, cerebellar in vivo pressure injection of BMY did not fully recover SS firing (Fig. 2J). This may be due to NE activation of α1_A_-, and α1_B_-ARs on GABAergic presynaptic terminals, leading to increased spontaneous inhibition of PC postsynapses [37]. BMY-7378 can also bind to α2_C_-ARs [71]. However, we precluded α2-AR involvement in dystonia formation, since Yoh application did not alleviate stress-induced dystonia (Fig. 1A), or rescued PC firing (Figure S6) [20]. More importantly, knockdown of specifically cerebellar α1_D_-ARs using shRNA completely abolished stress-induced dystonia without visible motoric side effects as seen in α1_D_ knockout mice and maintaining PC viability (Fig. 5) [72].

The mechanism, how blockade of α1-ARs prevents stress-induced attacks of motor dysfunction was recently postulated [73, 74]. Snell et al. showed that NE binding to α1-ARs results in a reduced open probability of small conductance K^+^ channels (SK) via phosphorylation of SK-associated calmodulin (CaM) by SK2-associated casein kinase 2 (CK2) [21]. Based on our study we demonstrated that the α1_D_-AR is the predominant adrenergic subtype driving stress induced dystonic episodes. However, the other α1AR subtypes may influence the phenotypic characteristics (i.e. onset, duration and severity) of stress induced dystonia. CaM functions as a Ca^2+^ sensor at the intracellular carboxy terminus of the SK channel, consequently CaM phosphorylation lowers its affinity to Ca^2+^, thereby reducing the open probability of SK2 channels [75, 76]. SK channels are also required for the intrinsic pacemaking activity in PCs [31, 77] and reduction of their activity through NE inhibition leads to further disruption of the already impaired PC firing in tottering^tg/tg^ mice (Figure S3A) [31, 78, 79], which strongly correlates to dystonia severity [35]. Interestingly, Snell et al. noted that CaM dephosphorylation is required for the recovery of PC firing regularity. This dysfunction in PC firing recovery is hypothesized to be much slower than the rise and fall of NE levels in the cerebellum and thereby dictating the duration of motor attacks [21]. Strikingly, we found that a small percentage of tottering^tg/tg^ mice could still display dystonia in the presence of BMY-7378 (Figs. 1C-E and 4), but were significantly milder and shorter, suggesting that BMY-7378 may have partially rescued dysfunctional PC firing as shown in our electrophysiological recordings (Fig. 2).

Our Ca^2+^ imaging data verified that BMY-7378 supports the PC firing frequency of ROI dendritic signaling close to those of control levels (Fig. 6). Imaging of PC dendrites showed large calcium transients, which are likely induced by inferior olive-climbing fiber inputs [55], suggesting that the measured calcium signals are produced by complex spikes. We showed that an increase in intra-dendritic Ca^2+^ correlates with the onset and formation of stress-induced dystonia in tottering^tg/tg^ mice. This was reflected in the increase of ΔF/F_0_ (approximation for maximal calcium entry into the dendrites) after dystonia started and increased integral (approximation of the total amount of Ca^2+^ influx) after vehicle injection (Fig. 6G, I), while ROI activity was not altered (Fig. 6E). This suggests that altered climbing fiber input and decreased complex spikes may contribute to the formation of stress-induced dystonia. Interestingly, we found that tottering^tg/tg^ mice display fewer CS compared to WT control mice (Figure S3) and that BMY-7378 helps to maintain CS formation after NE-induced inhibition (Figure S5G). We and others hypothesize that alterations in cerebellar calcium dynamics induce dystonia. A supportive study showed that dystonia induced by the depletion of type 1 IP_3_Rs from the cerebellum and brainstem was diminished by inactivating the cerebellum and inferior olive and the absence of PCs, indicating the involvement of the cerebellar inferior olive-climbing fiber circuitry in dystonia [80]. Intraperitoneal injection of BMY-7378 did not improve the formation of CS in our electrophysiological recordings when given after NE (Figure S5), but the ROI activity during the calcium imaging was decreased almost to WT levels, suggesting a stabilizing effect on the calcium dynamics (Fig. 6D). In addition, it is possible that BMY-7378 diminishes the release of intracellular Ca^2+^, as it impedes the activation of the Gq-PKC-IP_3_ pathway stimulated by α1-ARs [56], which normally results in release of Ca^2+^ from intracellular stores through IP_3_R and RyR. However, a potential contribution of intracellular Ca^2+^ needs to be determined in future experiments and is only hypothetical for now. We want to empathize here that several neurotransmitters and receptors have been implicated in formation of stress-induced dystonia in tottering^tg/tg^ mice, including 5-HT_2a_-R, NMDA and RyR [9, 11, 81]. Antagonism of both NMDA receptors and RyR was shown to prevent stress-, and caffeine-induced dystonia by preventing Ca^2+^ entry into the PC. Here we verified that the release of intracellular Ca^2+^ increases during the onset of stress-induced dystonic episodes in tottering^tg/tg^ mice, contributing to a novel valuable finding in understanding the NE effects on PC firing.

In accordance with previously published studies, our data strengthen the current understanding of NE and the role α1-AR in stress-induced dystonic episodes. In the present study, we demonstrated that cerebellar α1_D_-ARs are an essential contributor to the formation to stress-induced dystonia in tottering^tg/tg^ mice by increasing PC susceptibility to NE. Purkinje cell firing rate and regularity are thought to encode motor coordination related information [82] and are especially sensitive to changes in currents through P/Q-type channels [31, 83]. Since PCs from tottering^tg/tg^ mice display ~ 40% reduction of Ca^2+^ influx through P/Q-type calcium channels [7] and signs of axonal damage [32], protecting the already altered firing properties by blocking α1_D_-ARs helps to maintain the firing encoded information and prevent stress-induced paroxysmal dystonia caused by aberrant PC firing [21, 31, 35]. However, since many different receptor types on both PCs and DCN have been shown to contribute to dystonia, it is also well possible that the integration of all afferent signals onto the cerebellar cortex (e.g. Purkinje cell) or DCN, including noradrenergic, serotonergic and glutamatergic signals determines dystonia formation during stress [9, 11, 21, 81].

The use of α1-AR antagonists as therapeutical treatment remains to be determined. Prazosin or silodosin, another FDA-approved α1-AR antagonist, are suggested as therapeutics to treat EA2 patients [21]. Especially prazosin was shown to cause headache, dizziness, palpitation and tachycardia [84, 85]. Development and further exploration of α1_D_-AR specific drugs may not only be beneficial for EA2 patients, but for all patients suffering from episodic neurological disorders where stress is a common trigger [74]. However, the therapeutic use of α1_D_-AR blockers, although a more specific target, still needs to be explored and closely examined.

## Materials and methods

### Animals and genotyping

Genomic *Cacna1a* mutant mice tottering^tg/tg^ (JAX stock #000544; B6.D2-Cacna1a^tg/J^; gift from Dr. C.I. de Zeeuw) were bred with Gad2-Cre mice (JAX stock #010802; Gad2^tm2(cre)Zjh^/J [86], to obtain tottering^tg/tg^/Gad2-Cre mice, respectively. The genomic background of the mice was determined by PCR of genomic tail biopsy. The tottering allele was amplified using the following primer: *tott* forward 5’ TTCTGGGTACCAGATACAGG 3’, *tott* reverse 5’ AAGTGTCGAAGTTGTGCGC 3’. For Cre recombinase identification, the primers were: cre fw 5’ ATTCTCCCACCACCGTCAGTACG 3’, cre rev 5’ AAAATTTGCCTG CATTACCG 3’.

Adult mice (3–8 months) were single housed in the laboratory on a 12 h light/dark cycle with *ad libitum* access to food and water. Experiments were performed during the wake cycle of the animals. The present study was carried out in accordance with the European Communities Council Directive of 2010 (2010/63/EU) for care of laboratory animals and approved by a local ethics committee (Bezirksamt Arnsberg) and the animal care committee of North Rhine-Westphalia, Germany, based at the LANUV (Landesamt für Umweltschutz, Naturschutz und Verbraucherschutz, Nordrhein-Westfalen, D-45659 Recklinghausen, Germany). The study was supervised by the animal welfare commission of the Ruhr-University Bochum. All efforts were made to minimize the number of mice used for this study.

### Drug administration

Stock solutions of adrenergic receptor antagonist prazosin hydrochloride (Sigma Aldrich, P7791), yohimbine hydrochloride (Sigma Aldrich, Y3125) and BMY-7378 dihydrochloride (Tocris, #1006) were prepared in distilled water (d_2_H_2_O) and stored as recommended by the manufacturer. Working solutions were prepared daily using sterile NaCl (vehicle) in doses of 10 mg/kg prazosin hydrochloride, 20 mg/kg yohimbine hydrochloride and 10 mg/kg BMY-7378 dihydrochloride. Mice were intraperitoneally (i.p.) injected 30 min prior to the tests, with each mouse receiving each drug at least once. Test days were followed by at least one recovery day. The DREADD ligand CNO (clozapine-N-oxide, HelloBio, #HB6149) was prepared in d_2_H_2_O and diluted in 2% sucrose in working concentrations of 5 mg/kg and administrated orally. Stock and working dilutions were stored at −20 °C for 14 days.

### Stress-induced dystonia

Stress-induced dystonic attacks were triggered by either changing the cage (cage change stress), transport of mice for 10 min (cage transport stress) or restraining the animals for 10 min (short restraint) and releasing them to a novel cage, according to previous methods [42, 43] which were shown to elicit attacks in tottering^tg/tg^ mice [9, 20, 21]. Mice were observed for the presence or absence of dystonic attacks for 40 min and onset and duration of episodic dystonia were noted. Since dystonia follows a characteristic progression [9, 10], attacks can be classified and compared, thus severity of dystonia was scored as described below. After expiration of this time or when dystonia was over, mice were returned to their home cages and given at least one day to recover.

### Dystonia characterization

To quantify presence of dystonia, the overall motor behavior of animals was observed. Dystonia severity was scored every 5 min until end of attack following a modified scale as previously published [25]. Briefly, 0 = normal motor behavior; 1 = slightly slowed and/or abnormal movements; 2 = mild impairments, limited ambulation; 3 = moderate impairments, limited ambulation when disturbed; 4 = severe motor impairments, almost no ambulation, sustained abnormal postures; 5 = prolonged immobility in abnormal postures, no ambulation.

Dystonic episodes were scored severe, when a median score ≤ 3.5 or higher was reached, otherwise it was scored mild (score ≥ 3). The median of dystonia severity was first calculated per mouse/time, then for all mice within a group. Corresponding graphs display the median and the 75% and 25% quartiles per time.

### Behavior experiments

To evaluate ataxia, motor-coordination and balance skills, 6 month old tottering^tg/tg^, and control tottering^−/−^ mice underwent a beam walk test, gait analysis, hang wire test, rotarod and vertical pole test according to previous methods [42, 43]. Mice received either a saline or 5 or 10 mg/kg prazosin hydrochloride (in NaCl) i.p. injection 30 min prior to testing. Mice were given one day to recover between tests and drugs.

#### Beam walk

To access balance skills and motor coordination, mice were trained to cross a 70 cm long, 1 cm wide beam in 60 cm height. The start was illuminated and mice trained to cross the beam to reach the dark shelter (20 cm^3^) on the other site. During training, mice were shown the shelter, then placed on the beam with increasing distances until the beam was crossed completely. Mice were given a rest day and tested the following day. Mice were placed at the start of the illuminated beam and the latency to move toward the house (Idle), time to cross the beam and slips of paws was recorded. A maximum period of 120 s was given to perform the test. Falls were noted and scored as 120 s. Each individual was tested 3x, and the average was calculated.

#### Gait analysis

To evaluate ataxia, mice underwent the gait analysis. Individuals were placed at the end of a 10 × 70 × 10 cm (width x length x height) plastic chamber and trained to walk to the other end, where a hole connects the box with their home cage. To obtain footprints, forepaws were painted red and hindpaws painted blue with non-toxic, water-soluble childrens paint (Pelikan) and the box laid out with white paper. The length and width between steps were measured by hand. Each mouse underwent one trial per condition.

#### Hangwire

As an indicator for grip and muscle strength, the hangwire test was performed. Mice were placed on a wire screen (12 mm x 12 mm grid) and hung upside-down above a 15.5 × 29 × 26.3 cm (width x length x height) box for a maximum of 60 s. The latency to fall was recorded. Mice that did not fall within the trial period were removed and given a 60 s score. Each mouse was tested twice and the average taken.

#### Rotarod

To screen for motor coordination and balance skills, mice underwent the rotarod test (Columbus Instruments). Therefore, mice were placed on a rod (3 cm) rotating at 4 rpm for 1 min (acclimatization phase), followed by a speed increase of 0.1 rpm/s to a max of 40 rpm. The trial was over when all mice fell, latency to fall and speed were recorded. Mice that fell during the acclimatization phase were placed back on the rod 3x, after that they received a score of 4 rpm and 0 s time.

#### Vertical pole test

Motor coordination and balance skills were accessed using the vertical pole test. A 52 cm long metal pole wrapped in tape for grip was secured to a platform. The pole was placed in an empty cage and the platform and floor covered in towels. Mice were positioned on top of the pole face upwards. Their latency to turn around and time to climb down the pole were recorded. A max of 120 s was given to complete the test. Mice falling were given the maximum time. Individuals were tested 3x and the average was taken.

#### Open field

Mice were placed in the center of a 50 x 50 cm opaque plastic box and allowed to freely explore for 5 min. Their exploring path was tracked using the EthoVisionXT 8.5 video tracking software (Noldus). The total distance moved (cm) and time spent in the center or border region of the arena were analyzed. The apparatus was cleaned between subjects with 70% ethanol. Each mouse underwent 1 trial. Statistical significance was analysed using SigmaPlot software (Systat Software Inc.).

#### Grip strength

Muscle strength in tottering^tg/tg^ mice was additionally examined using a grip strength meter (Ugo Basile^®^, Gemonio, Italy) either 30 min after BMY-7378 or vehicle injection (i.p.) or 4 weeks after sh-RNA injection. The mouse was allowed to grab a T-shaped bar with their front paws, then gently pulled back at their tail. The force and time until the mouse released the bar was measured. Each mouse was tested three times, and the mean was calculated per mouse.

### QPCR

Quantification of adrenoreceptor expression tottering^tg/tg^ (*n* = 10) and tottering^−/−^ (*n* = 10) 3–4 months old mice were deeply anaesthetized and quickly decapitated. Cerebella were immediately removed and frozen in liquid nitrogen. RNA isolation was conducted using the ReliaPrep™ RNA Tissue Miniprep System (Promega) and concentration (ratio A260/A280) and quality (ratio A260/A230) of RNA were estimated using a NanoDrop 2000c Spectrophotometer (Thermo Fisher Scientific). cDNA synthesis was performed using the RevertAid First Strand cDNA synthesis kit (Thermo Fisher Scientific). For qPCR, probes were amplified in triplicates using the SYBR green based GoTaq^®^ qPCR Mastermix (Promega) in a Rotor-Gene Q real-time PCR cycler (Qiagen) in a two-step program (40 cycles: 15 Sect. 95 °C, 90 Sect. 60 °C). Obtained ΔCT values were analyzed with the corresponding Rotor-Gene Q Series Software. Normalization was performed through geometric averaging of multiple reference genes [87], previously selected for cerebellar expression stability [88] and confirmed with the NormFinder software [89]. Primer specificity was verified via melting curve analysis and gel electrophoresis. All primers were obtained from Invitrogen (Germany) and are listed in table S8.

#### Analysis of sh-RNA induced α1_D_-KO

Both tottering^tg/tg^ injected with either sh-[ADRA1D] (*n* = 9) or sh-[scramble] (*n* = 5) were sacrificed as mentioned above. One hemisphere was used for qPCR analysis; the other half was used for WB analysis.

### Histology

Mice were deeply anesthetized with ketamine and xylazine (100 mg/kg and 20 mg/kg, respectively) and perfused transcardially with 1x PBS followed by ice-cold 4% PFA (paraformaldehyde, Sigma-Aldrich) in PBS (pH 7.4). Brains were dissected and post-fixed for 1 h in 4% PFA, then cryoprotected in 30% sucrose in PBS overnight before slicing using a cryostat (Leica CM3050 S).

#### Adrenoreceptor staining

35 μm sagital slices were co-stained with mouse-α-calbindin (1:300, Sigma, C9848). Sections for staining with rabbit-α-α1_D_ (1:200, Alomone labs, AAR-019), rabbit-α-α2_A_ (1:100, Alomone labs, AAR-020), and rabbit-α-α2_B_ (1:100, Alomone labs, AAR-021), were caught and washed in 1 x PBS, followed by blocking in 10% NDS in 0.3% PBST and incubation in primary antibodies at 4 °C overnight. Sections for rabbit-α-α1_A_ (1:250, Invitrogen™, PA1-047), rabbit-α-α1_B_ (1:100, Alomone labs, AAR-018), and rabbit-α-α2_C_ (1:100, Alomone labs, AAR-022) were caught and washed in 1 x TBS, blocked in 10% NDS in 0.25% TBS-T before overnight incubation of primary antibodies in blocking solution at RT. The next day, slices were washed and incubated 3 h at RT in secondary antibodies donkey-α-rabbit DyLight 650 (1:500, SA5-10169, Thermo Fisher) and donkey-α-mouse Alexa 568 (1:500, Invitrogen™, A10037) in blocking solution, respectively, before mounting. Fluorescent Nissl staining (NeuroTrace™ 435/455, Invitrogen™ N21479) was conducted before blocking.

#### Calbindin staining

For Purkinje cell identification of GCaMP8m injected cerebella, a calbindin staining was conducted using chicken-α-*calbindin (1:500*, antibodies.com, *A85359)*. 25 μm thick sections were washed 3x in 1x PBS, followed by blocking in 10% NDS in 0.5% PBST and incubation in primary antibody at 4 °C overnight. The next day, slices were rinsed in 1x PBS, then incubated in donkey-α-rabbit DyLight^®^ 650 (1:500, SA5-10169, Thermo Fisher) and donkey-α-chicken Cy3 (1:500, AP194C, Merck) in blocking solution for 2 h at RT.

#### Aldolase C staining (Zebrin II)

To verify sh-RNA expression in both Zebrin II^+^ and Zebrin II^−^ PCs, 35 μm thick sections were washed 3x in 0.5% PBS-T, then blocked in 10%NDS in 0.5% PBS-T for 2 h. Sections were then incubated 1:300 rabbit-α-Aldolase C (Invitrogen, PA5-27659) in blocking solution at 4 °C overnight. The next day, slices were rinsed in 3x in 0.5% PBS-T, then incubated in donkey-α-rabbit DyLight^®^ 650 (1:500, ab96894, abcam) for 2 h at RT.

### Imaging

All images were obtained using an inverted Leica TCS SP5 confocal laser scanning microscope (Leica DMI6000 B, Wetzlar Germany) interfaced to a computer running the Leica Application Suite Advanced Fluorescence software (LAS AF 2.6). Sequential scans were performed according to the required wavelengths using 20x or 40x objectives. Sequential z-stacks of 10 to 15 images were made for each section and crosstalk of fluorophores was eliminated automatically by the LAS AF 2.6 software. Acquired images were analyzed using ImageJ (NIH).

### Electrophysiology

#### Recordings

Electrophysiological recordings were conducted as previously described [90]. Briefly, mice were deeply anesthetized with 2% isoflurane via a precision vaporizer (E-Z Anathesia, Euthanes Corp, Palmer, PA, USA) and placed in a stereotaxic frame (SR-6 M, Narishige, Tokyo, Japan). Mice received 2 mg/kg rimadyl s.c. and 2% lidocaine hydrochloride locally before surgery. A craniotomy (2 × 2 mm) was performed above the recording site, approximately – 6.5 mm from bregma using a dental drill. The dura mater was removed and extracellular recordings of PCs were performed using a multielectrode system (Eckhorn system, Thomas Recording, Giessen, Germany) with 5 electrodes (impedance, 2–3 MΩ, at 1 kHz, Thomas Recording) simultaneously. Signals were amplified and filtered (band-pass, 0.1–8 kHz) with a multichannel signal conditioner (CyerAmp380, Axon Instruments, Union City, CA, USA) and sampled with 32 kHz via a A/D converter (NI PCI-6259 multifunction data acquisition board, National Instruments, Austin, TX, USA), controlled via a custom-made software using MatLab (MathWorks, Natick, MA, USA) as previously presented [90]. For microinjection of adrenoreceptor modulators diluted in ACSF, quartz glass microinjection pipettes (outer diameter: 115 μm, inner diameter: 85 μm; Thomas Recording, Giessen, Germany) were positioned next to the recorded cell. Via pressure injection, norepinephrine (20 mM, Sigma Aldrich, A7257) was applied after 150 s of reference recording and its effects recorded for 150 s before application of the antagonists was conducted (1 mM yohimbine hydrochloride, α2- AR antagonist, Sigma Aldrich, Y3125; 1 mM prazosin hydrochloride, α1-AR antagonist, Sigma Aldrich, P7791; 1 mM BMY-7378 dihydrochloride, α1D-AR antagonist, Tocris, #1006). Recordings were conducted for 6 min and saved for offline analysis.

#### Data analysis

Single unit action potentials were detected with custom-made software implemented in Matlab [90]. Purkinje cells were identified by their unique firing properties. Simple spikes occur spontaneously at a frequency of 20–150 Hz, while CS were observed with < 1 spikes/sec, characterized by a strong depolarization spike and multiple wavelets. Cells were analyzed, when a CS was followed by a SS pause of ~ 25 ms, proving spikes were generated by one single PC. The mean firing rate of both SS and CS within the 150 s recording intervals was compared. In addition coefficients of variation (CV) of interspike intervals (ISIs) *[CV = stdev (ISI)/mean(ISI)]* and the coefficients of variation for adjacent intervals CV2 of *ISIs [CV2 = 2 | ISIn + 1–ISIn |/(ISIn + ISIn + 1)]* were calculated. An average of CV2 over n estimates the intrinsic variability of a spike train, nearly independent of slow variations in average rate. Error bars denote SEM.

### Chronic cerebellar drug infusion

Chronic perfusion of the cerebella of tottering^tg/tg^ mice was performed using the *ALZET*^®^ osmotic pumps (model 1007D, Durect), connected to the ‘Brain Infusion Kit 3’ (#0004760, Durect). Pumps were prepared sterilely according to the manual. Briefly, the vinyl catheter tubing was cut to ~ 3 cm length, connected to the infuser and 2 spacer discs were glued to the infusion cannula to allow tissue penetration of ~ 1.5 mm depth. Pumps, catheter and the infuser were filled with either 1 mM BMY in ACSF or ACSF alone before connecting all parts. 0.01% methylene blue was added to solutions to allow for postmortem identification of the perfusion site. For osmotic equilibration, assembled pumps were incubated overnight at 37 °C in 0.9% NaCl. The next day, mice were prepared for surgery as already described and a craniotomy was drilled at AP: −6.5 mm from bregma. The osmotic pump was inserted into a lateral subcutaneous pocket of the animal, while the infusion canula was inserted into the craniotomy (AP: −6.5 mm, ML: 0 mm, DV: −1.5 mm) and fixed to the skull using the light-cured Gradia^®^ DIRECT Flo composite (GC corporation). Mice were placed in their home cages and monitored closely for recovery. Carprofen (100 µl/100 ml) was orally administrated for 5 days. The cage change stress test paradigm was performed 5-, 7-, 10- and 14 days post implantation to monitor frequency and severity of stress-induced dyskinesia.

### Intracerebellar virus injections

Mice received subcutaneous injection of carprofen (2 mg/kg) for analgesia 30 min prior to surgery, then deeply anesthetized with 1.5–2.0% isoflurane and placed into a stereotactic frame (Narishige, Japan). OmniVision^®^ gel was applied to the eyes to prevent dehydration. 2% lidocaine hydrochloride was subcutaneously injected at the scalp for local anesthetic. The skin was disinfected and opened with a sagittal incision along the midline. Craniotomies were performed above the CB vermis according to the methods of Bohne et al. [91]. Briefly, the virus was sucked into a customized glass pipette attached to a 5 ml syringe. Via pressure injection, the virus was dispensed in 50–100 μm steps with a time interval. After injection, the skin was sutured (Surgicryl Monofilament, Belgium) and mice were removed to their home cages. Analgesia was applied for 5 subsequent days or longer when required and animals were given 14–21 days to recover from surgery.

### ShRNA mediated KO of α1_D_-ARs

shRNA sequences against *Mus musculus* Adra1d were obtained from VectorBuilder in AAV2-eGFP-U6-shRNA packed adeno-associated viruses. AAVs used included the following: AAV2-CMV-eGFP-U6-mAdra1d[shRNA#1] at a titer of 3.62 × 10^13^ GC/ml; AAV2-CMV-eGFP-U6-mAdra1d[shRNA#2] at a titer of 4.04 × 10^13^ GC/ml; AAV2-CMV-eGFP-U6-mAdra1d[shRNA#3] at a titer of 4.92 × 10^13^ GC/ml and AAV2-CMV-eGFP-U6-Scramble at a titer of 1.20 × 10^13^ GC/ml. 3–4 months old tottering^tg/tg^ and tottering^−/−^ mice of both sex were injected at the following injection sites: AP: −6 mm, ML: 0 mm, DV: −1.2 – −0.9 mm; AP: −6 mm, ML: ±1. 8 mm, DV: −2 – −1.7 mm; AP: −6.25 mm, ML: 0 mm, DV: −1.2 - −0.9 mm; AP: −6.25 mm, ML: ± 1 mm, DV: −1.7 - −1.4 mm; AP: −6.4 mm, ML: 0 mm, DV: −1.2 – −0.9 mm; AP: −6.4 mm, ML: ± 2.7 mm, DV: −2 - −1.7 mm; AP: −6.6 mm, ML: 0 mm, DV: −1.2 – −0.9 mm; AP: −6.6 mm, ML: ± 1.5 mm, DV: −1.7 - −1.4 mm; AP: −6.96 mm, ML: 0 mm, DV: −1.2 – −0.9 mm; AP: −6.96 mm, ML: ± 1 mm, DV: −1.7 - −1.4 mm. This was needed for our paradigm to infect the whole cerebellum. Starting 3 weeks post injection, animals underwent a weekly cage change for 3 weeks. After a total of 6 weeks of expression, mice were killed through cervical dislocation. Their cerebellar were removed and bisected. Hemispheres were individually used for qPCR or WB analysis. One shRNA injected mouse was perfused for verification of virus expression. 100 μm thick vermal sections were taken of one scramble injected mouse using a vibratome (Leica) prior to analysis.

### Western blots

Cerebellar hemispheres were weighted then lysed and homogenized in TDL buffer (1 M HEPES buffer (pH 7.4), 5 M NaCl, 0.5 M EDTA (pH 8), 10% NP40, 10% natrium deoxycholate, 10% SDS, 25x protease inhibitor). Lysates were incubated on ice for 30 min, then mixed and centrifuged at 12,000–14,000*g for 10 min at 4 °C. Protein concentration was determined through photometric OD_600_ using a Bradford protein assay 5 min after lysis. Equal amounts of Laemmli sample buffer were added to the supernatants and cooked for 5 min at 99 °C. Samples were aliquoted and stored at −20 °C. Samples were loaded onto 12% T Tris-HCl separation gels covered by 4% T collecting gels and run for 55–77 min, 20 mA. Protein transfer onto a PVDF membrane was conducted for 1.5 h at 200 mA in running buffer.

#### Antibody incubation

PVDF membranes were incubated in blocking buffer (3% milk powder, 2% BSA in 0.1% TBS-T) for 1 h, at RT on a shaker prior to primary antibody incubation (see table S7) over night at 4 °C. Membranes were washed 3 × 5 min in 0.1% TBS-T and incubated in secondary antibodies (see table S7) for 1.5 h at RT. Membranes were washed again prior to detection.

#### Detection

Detection was performed using the ECL™ Select Western Blotting Detection Reagent (Amersham™). Equal amounts of the two detection reagents were mixed and added to the PVDF membranes. Incubation was performed for 5 min at RT before transferring the PVDF membranes to a clear plastic cover and immediately placed into a Hypercassette™ (Amersham Pharmacia Biotech, Inc.). Under red-light conditions, a medical X-Ray film (Fujifilm Super RX-N) was placed on the membranes and exposed for 30 s to 2 min depending on the intensity of the bands. X-Ray films were developed in developer solution (AGFA Dentus D-1000, Kulzer) for 2 min, washed in water, and fixed in fixer solution (AGFA Dentus F-1000, Kulzer) for 2 min. After that, the films were rinsed in water and dried hanging in a ventilated cabinet at RT. X-Ray films were scanned and band intensity analysed using ImageJ (Fiji).

#### Stripping

Protein loaded PVDF membranes were used two times. Therefore, the old primary antibody was removed by initially washing in 0.1% TBS-T for 15 min, followed by incubation in ROTI^®^ Free Stripping buffer 2.0 (Carl Roth) for 30 min at RT. Membranes were washed twice in 0.1% TBS-T for 20 min, then blocking was repeated.

### In vivo Ca^2+^ imaging of Purkinje cells

#### Implantation

Mice were injected with AAV-DJ/2-mCaMKIIα-jGCaMP8m-WPRE-bGHp(A) (#p630, VVF, Zurich) in the cerebellar vermis at AP: −6.25 mm, ML: ± 0 mm, DV: −1.2 & 0.9 mm as already described and given 14–21 days for virus expression and recovery. Then, mice were implanted with the ProView™ Prism Probe (1.0 mm diameter, ~ 4.3 mm length, #1050–004606, Inscopix, USA). Briefly, mice were anaesthetized and fixed to the stereotaxic frame. The skull was primed using OptiBond™ Universal adhesive (#36519, Kerr, Germany) and 1/3 of a screw (2 mm length) was drilled into the skull above the motor cortex for more stability of the implant. A craniotomy (~ 1.8 mm diameter) was drilled above the injection site using a trephine (FST, #18004-18), the exposed cerebellum was moisturized with ACSF and slowly cut ~ 200 μm deeper than the virus was injected using a micro knife (FST, #10315-12) either from anterior to posterior for ML imaging experiments, or from medial to lateral for AP imaging experiments. At the cutting edge, the prism was slowly lowered to the desired depth (~ 200 μm deeper than virus was injected) and attached to the skull using light-cured Gradia^®^ DIRECT Flo composite (GC corporation). Mice recovered for 2 weeks before weekly assessment of virus expression and Ca^2+^ signaling before attachment of baseplates (#1050–004638, Inscopix, USA) above the lens using Gradia^®^ DIRECT Flo. Attachment of baseplates was conducted under visual control where the miniscope was slowly lowered towards the lens using the micromanipulator to allow for optimal positioning and distance of the miniscope to the lens for maximal Ca^2+^ signals. Afterwards, the protective cap was placed on the baseplate to protect the lens from scratches, dust or animal bedding that could potentially contaminant the lens. Animals were given at least 2 days to recover from anesthesia before habituated to handling and mounting the miniscope.

#### Imaging

Mice were habituated to handling and mounting of the nVista 2.0 Miniscope Sytem for 2 days. On recording days, mice were injected i.p. with saline or 5 mg/kg BMY-7374 30 min prior to recordings to explore alterations in Ca^2+^ signals of PCs before, during and after stress-induced dystonia. The miniscope was secured to the baseplate and the cage change stress paradigm was conducted. Recordings of calcium transients were acquired using the nVista Inscopix Data Acquisition Software (IDAS, Inscopix, USA) at 30 Hz, LED power 1–1.3, Gain 3–4). Videos were acquired for 1 min per video in 5 min intervals for a total of 40 min or until end of the dystonic attack. The behavior of mice during the calcium recording acquisition was simultaneously recorded using a USB video camera connected to the laptop to allow for synchronization of behavior and Ca^2+^ signals of mice. Sessions were individually saved and stored on an external hard drive for analysis. Each mouse was recorded several times. Only one recording was performed per day/mouse with 2 day intervals between recordings.

#### Preprocessing 

Analysis of Ca^2+^ recordings was performed using the Inscopix Data Processing Software (IDPS, Inscopix, USA). Videos were cropped, temporally down sampled and motion corrected, before ΔF/F_0_ was acquired to normalize each pixel value in the recording to a baseline level. Since Ca^2+^ signals varied between recorded mice, individual regions of interest (ROIs), putative PC dendrites were manually encircled and analyzed for their signals. Signal traces were deconvolved and exported for offline analysis as csv files.

#### Data analysis

Exported traces were further analyzed and plotted using a custom-made software implemented in Matlab (Mathworks). This software calculated the spikes per second (ROI activity), integral of Ca^2+^ peaks and Ca^2+^ peak height (ΔF/F_0_), CV1 and CV2, which were exported into SigmaPlot. Fluorescence signals indicating calcium transients of individual ROIs were compared within the recorded videos of one session per mouse.

### Virus production

Adeno-associated viruses were either purchased or produced as previously described [91]. Briefly, low passage 293 T cells were co-transfected with pAAV-EF1α-DIO-hM4Di_(Gi/o)_-mcherry (original plasmid pAAV-hSyn-hM4D(Gi)-mCherry was a gift from Bryan Roth (Addgene plasmid #50475; http://n2t.net/addgene:50475; RRID: Addgene_50475)), pAAV-RC9, and pHelper using the polyethylenimine (PEI) based protocol. Three days after transfection, cells were harvested, pelleted (3,700 g, 20 min, 4 °C), resuspended in 10 ml lysis buffer (150 mN NaCl, 50 mM Tris-HCl, pH 8.5) and lysed via seven freeze (15 min)/thaw (10 min) cycles in a dry ice/ethanol and 37 °C water bath. The cell suspension was treated with DNase I (Roche) for 30 min at 37 °C to degrade free DNA. The cell debris was spun down at 3,700 g for 20 min at 4 °C. The supernatant was collected in a syringe and filtered into a 15 ml falcon tube through a 0.2 μm filter to obtain the crude lysate. Then the supernatant was resuspended in a polyethylene glycol (PEG) solution overnight at 4 °C and pelleted at 3,700 g for 20 min at 4 °C. The pellet was resuspended in PBS, 0.001% pluronic and aliquots were stored at −80 °C until further use.

### In vivo chemogenetic manipulation

For chronic silencing of the MLIs, 5 mg/kg CNO in 2% sucrose (to mask bitterness) was orally administrated *ad libitum* for at least 5 days prior to testing as previously described [39, 40]. Bottles were weighed daily to control sufficient intake of CNO. Solutions were replenished as needed or every third day. Mice underwent the stress-induced dystonia tests after 5 days of consumption of either 2% sucrose, or 5 mg/kg CNO in 2% sucrose. One test per day/mouse was performed and stress tests were conducted in random order, though each mouse underwent each test under each condition three times.

### Statistical analysis

Test procedure, statistical significances and number of animals (*n*) for each experiment can be found in table S1-S3 and S7-S10. Statistical analysis was conducted with SigmaPlot (Systat Software), the level of significance was set to *p* < 0.05. Error bars display mean ± SEM, if not stated otherwise Statistical significance is reported as n.s (not significant); * *p* < 0.05; ** *p* < 0.01; *** *p* ≤ 0.001.

## Supplementary Information

Below is the link to the electronic supplementary material.

Supplementary Material 1 (DOCX 7.97 MB)

Supplementary Material 2 (MP4 36.3 MB)

Supplementary Material 3 (MP4 25.7 MB)

Supplementary Material 4 (MP4 42.5 MB)

Supplementary Material 5 (MP4 23 MB)

## Data Availability

Data needed to evaluate the conclusions in the paper are present in the paper and/or the Supplementary Materials. For additional information, please contact the corresponding author at melanie.mark@rub.de.
