# Supplementary material for: Cerebellar α1D- adrenergic receptors mediate stress-induced dystonia in totteringtg/tg mice
Source: Cell Mol Life Sci. 2025 Oct 6;82(1):344. doi: 10.1007/s00018-025-05843-1 (PMC12500514; doi:10.1007/s00018-025-05843-1)
Supplement: Supplementary file 1 — Supplementary Material 1 (DOCX 7.97 MB) [file 18_2025_5843_MOESM1_ESM.docx]

Supplementary Materials for

**Cerebellar α1_D_-adrenergic receptors mediate stress-induced dystonia in tottering^tg/tg^ mice**

Pauline Bohne, Mareike Josten, Lina Rambuschek, Jana Brüggemann, Xinran Zhu, Max O. Rybarski and Melanie D. Mark

*Corresponding author. Email: melanie.mark@rub.de

**This PDF file includes:**

Figs. S1 to S8

Tables S1 to S10

Movies S1 to S4

**Other Supplementary Materials for this manuscript include the following:**

Movies S1 to S4


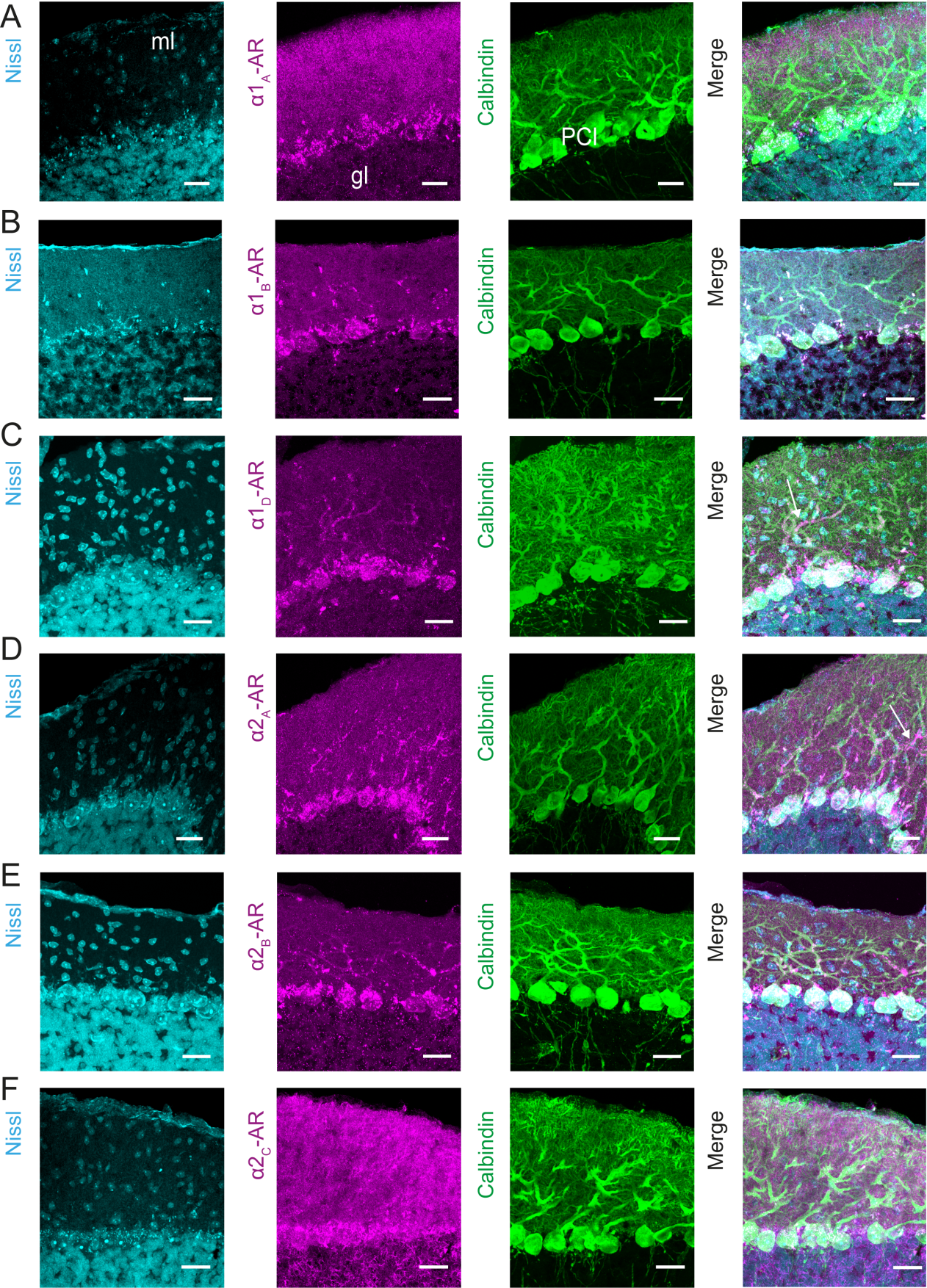


Fig. S1. Adrenergic receptor localization in the cerebellar cortex of tottering^tg/tg^ mice.

**(A)** IHC staining against α1_A_-AR (magenta) shows a punctual expression in the molecular layer and PC soma in the cerebellum of tottering^tg/tg^ mice. Both the α1_B_- **(B)** and α1_D_-AR **(C)** are predominantly located on PC soma. **(D)** IHC staining against the 2_A_-AR shows strong labeling of PC soma and dendrites (magenta), as indicated by overlay with calbindin staining (green). **(E)** Overlay of calbindin and α2_B_-AR staining reveals that the α2_B_-AR is located on cerebellar PCs. **(F)** IHC staining against the α2_C_-AR shows expression in the ml, PCl and gl. Scale bars 25 µm. AR = adrenergic receptor, gl = granular layer, ml = molecular layer, PCl = Purkinje cell layer


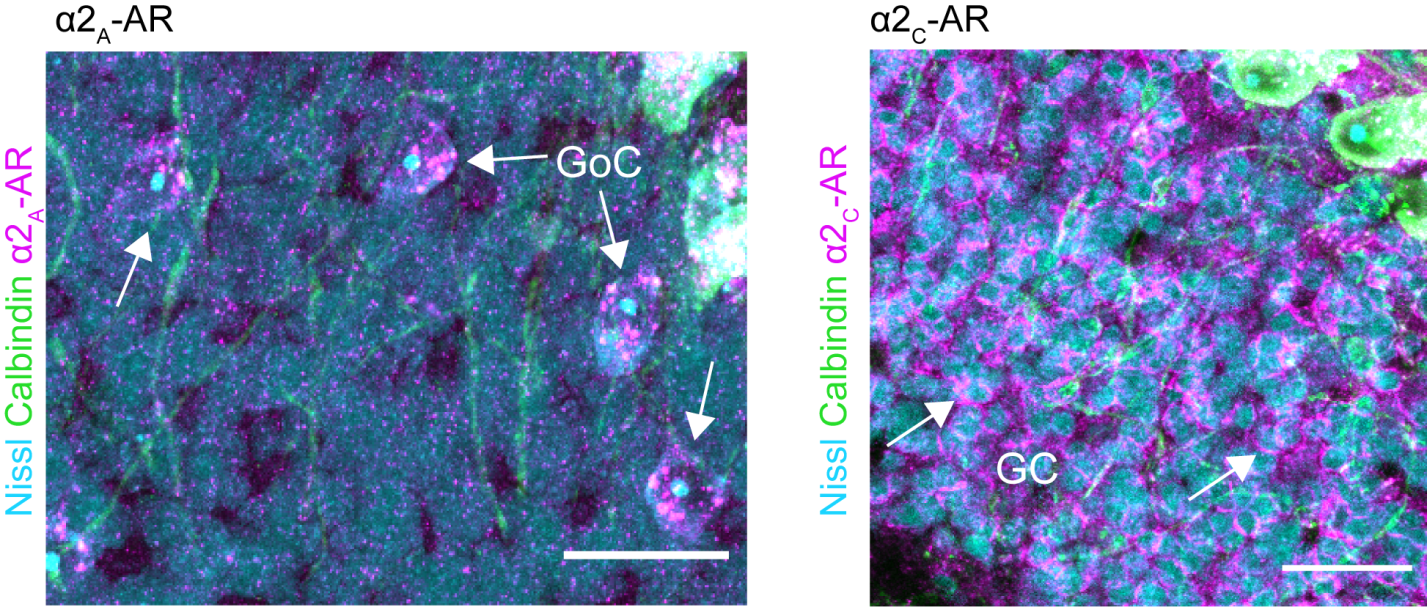


Fig. S2. α2_A_ and α2_C_ adrenergic receptors are located on cerebellar Golgi cells (GoC) and granule cells (GC).

Antibody staining against the adrenergic α2_A_ receptor subtype indicates localization on interneurons in the granular layer (gl), potentially Golgi cells (GoC). Staining against the α2_C_-AR shows membrane-like staining around cerebellar granule cells (GC), potentially showing presynapses. Scale bars 25 µm. AR = adrenergic receptor


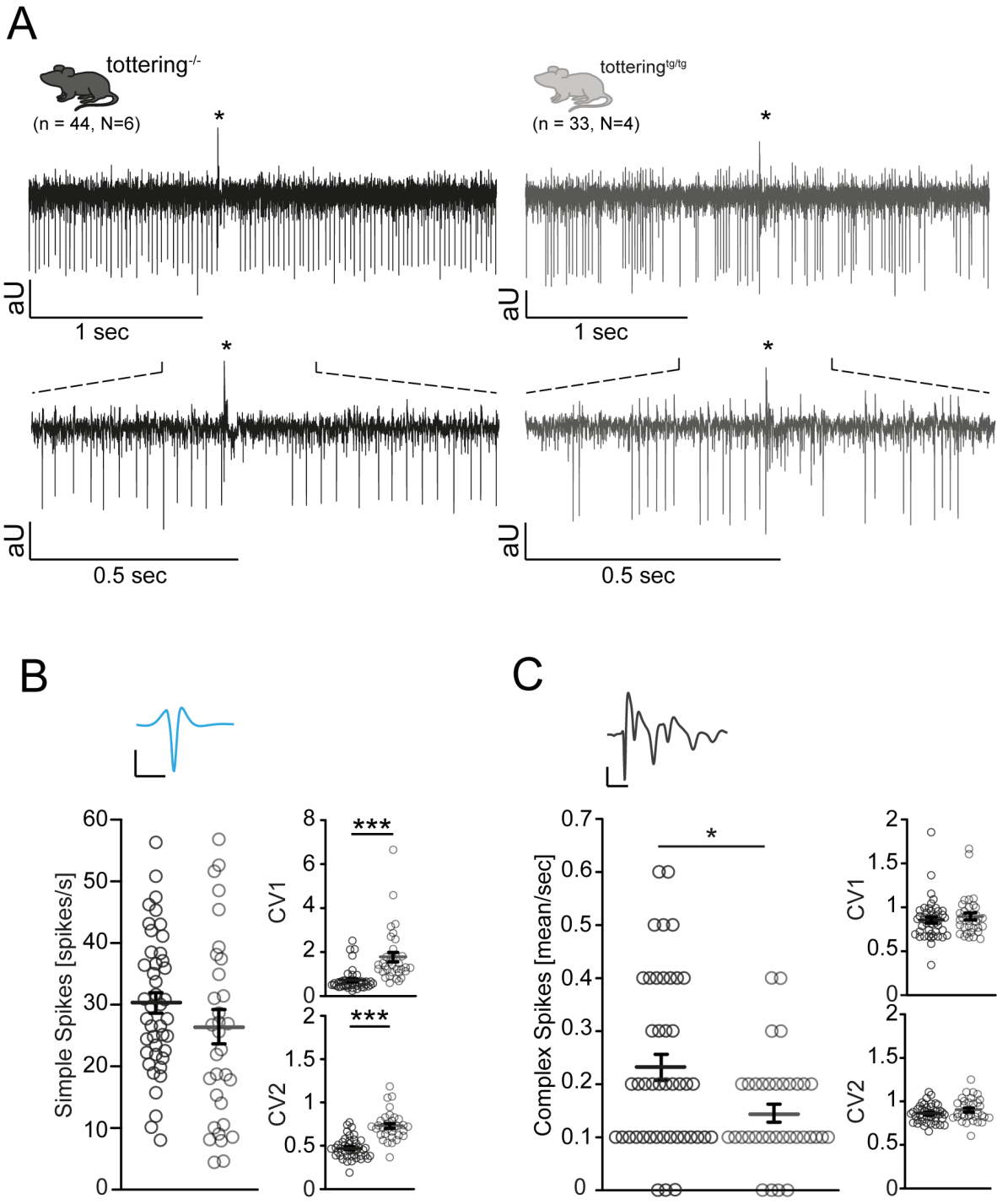


Fig. S3. Purkinje cell firing is irregular in tottering^tg/tg^ compared to control tottering^-/-^ mice.

**(A)** Example traces from extracellular recorded PCs in tottering^-/-^ and tottering^tg/tg^, mice displaying the irregularity in PC firing of tottering^tg/tg^, indicating complex spikes (*). **(B)** Mean simple spike firing is not altered in tottering^tg/tg^ mice, but are highly irregular and variable. **(C)** The mean number of complex spikes is decreased in tottering^tg/tg^ compared to control mice, while there is no difference in regularity and variability indicated by CV1 and CV2 values. Data are presented as mean±SEM. Statistical significance was evaluated by students t-test. (*p≤0.05, **p≤0.01, ***p≤0.001).


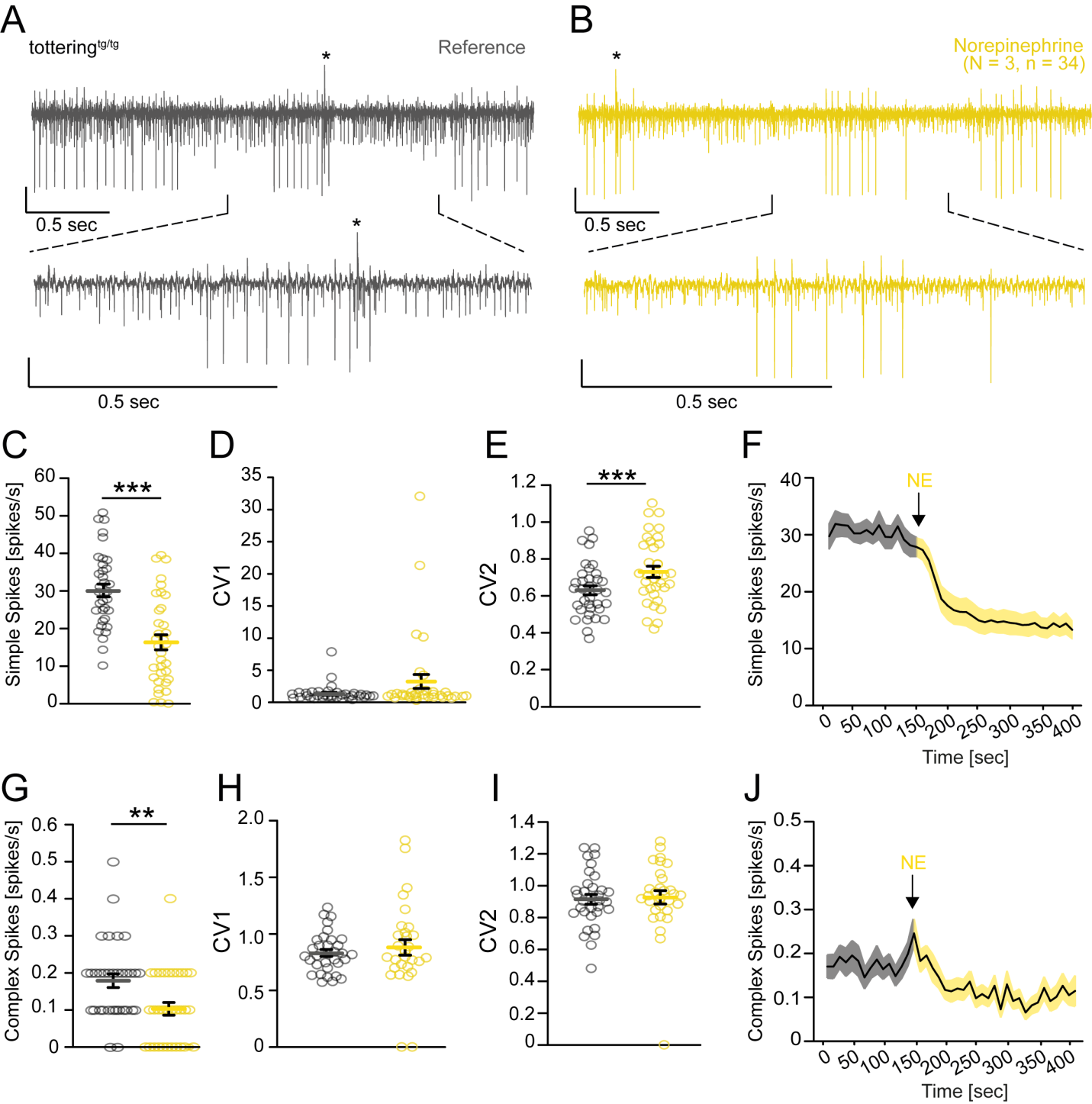


Fig. S4. Extracellular *in vivo* recordings verify inhibitory effects of NE on tottering^tg/tg^ Purkinje cells.

Example traces of an extracellular recorded PC of a tottering^tg/tg^ mouse before **(A)** and after **(B)** exogenously applied NE. **(C)** Simple spikes are significantly decreased after NE application. **(D)** CV1 showed a trend to be increased, while CV2 was significantly increased **(E)**. **(F)** Course of the mean simple spike firing of all PCs recorded displayed that the firing frequency does not recover after NE application during the recording period of 400 sec. **(G)** Complex spike firing was significantly decreased in tottering^tg/tg^ mice after NE application. **(H)** The CV1 of complex spikes tended to be increased, while the CV2 was not altered **(I)**. **(J)** Course of the mean complex spike firing of all PCs recorded displayed that the firing frequency did not recover after NE application during the recording period of 400 sec. Data are presented as mean±SEM. Statistical significance was evaluated by paired t-test. (*p≤0.05, **p≤0.01, ***p≤0.001). See Table S8 for exact p-values and mean.


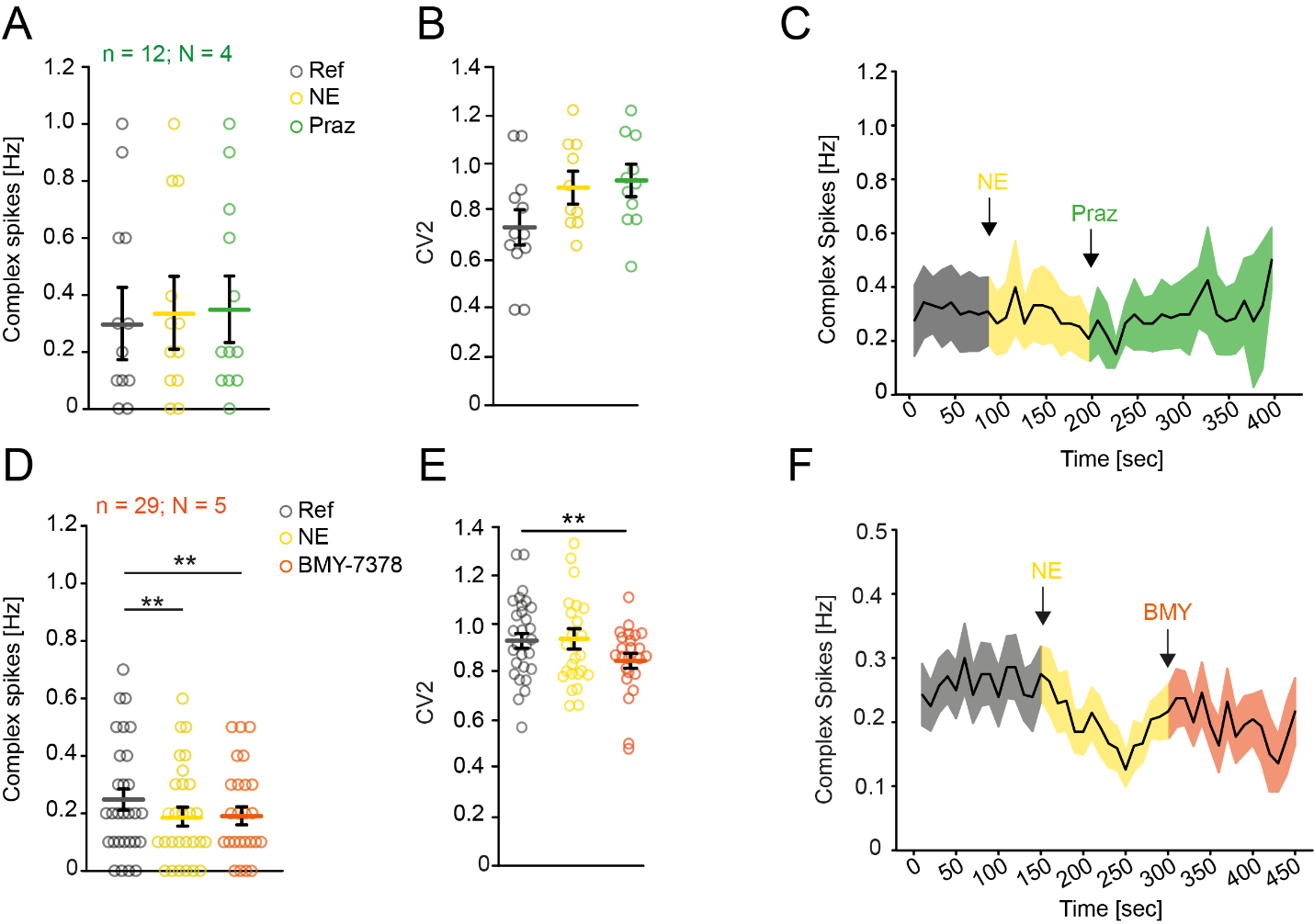


Fig. S5. Effects of α1-AR antagonists Prazosin and BMY-7378 on Purkinje cell complex spikes in tottering^tg/tg^ mice *in vivo*.

**(A)** Praz does not impact complex spike firing after NE application. **(B)** The CV2 was not altered after NE and Praz application. **(C)** Course of the mean complex spike firing of all PCs recorded shows a slight recovery after Praz application. **(D)** Complex spikes are decreased after NE pressure injection and not recovered with BMY-7378 administration, but the intrinsic complex spike variability of recorded PCs is improved after BMY-7378 application **(E)**. **(F)** Course of the mean complex spike firing of all PCs recorded after NE and BMY-7378 application. Data are presented as mean±SEM. Statistical significance was evaluated by paired t-test. (*p≤0.05, **p≤0.01, ***p≤0.001).


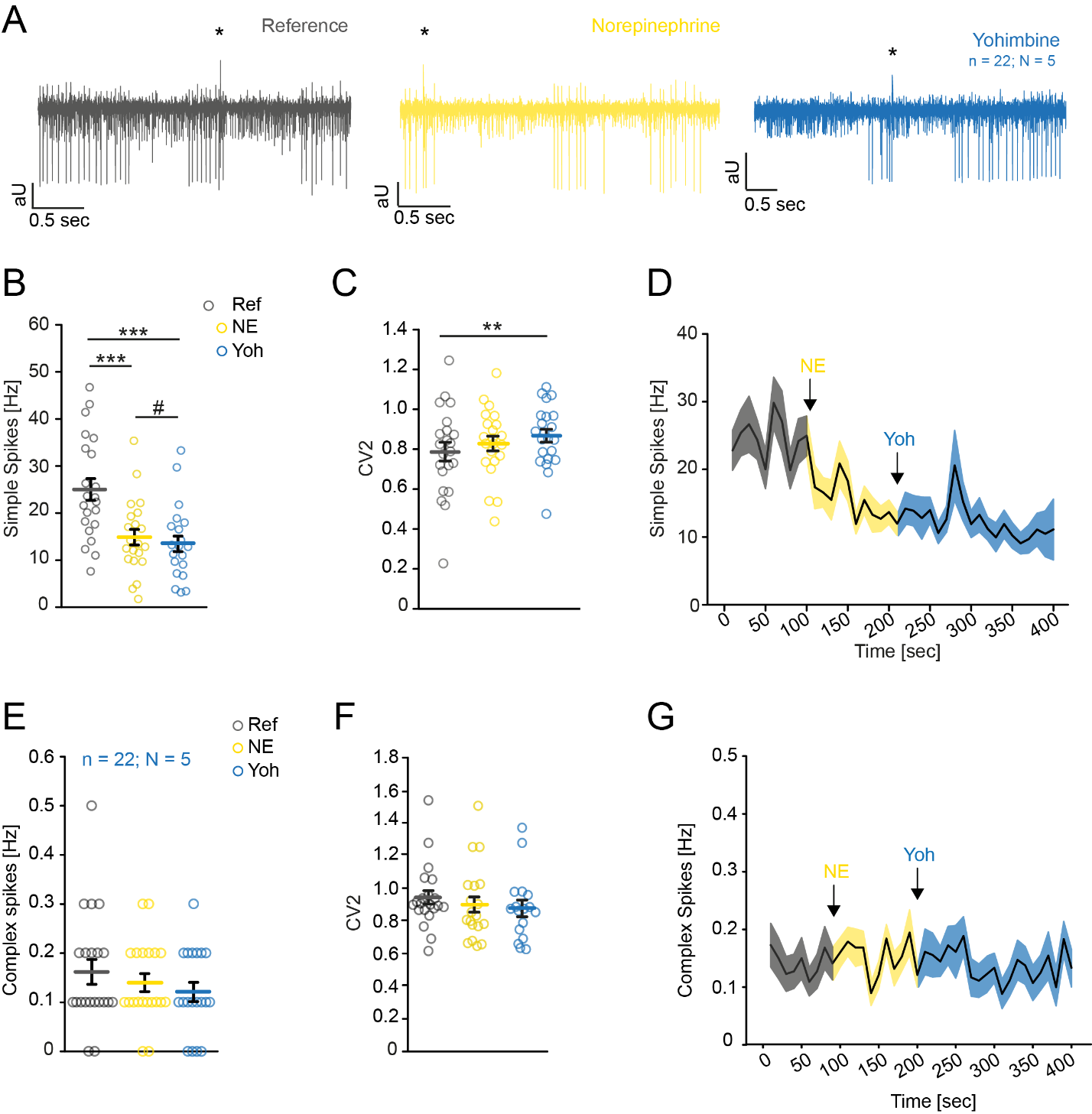


**Fig. S6: Pharmacological blockade of cerebellar α2-ARs does not recover PC SS or CS. (A) E**xample traces of reference PC SS spontaneous activity (grey), after NE application (yellow) and Yoh application. **(B)** The α2-AR blocker YOH did not recover PC SS firing after NE-mediated inhibition (14.882±1.657 vs 13.464±1.642, p=0.055)**. (C)** The irregularity increased after Yoh application compared to reference recordings as indicated in the CV2 (0.788±0.0468 vs 0.868±0.0322, p=0.006). **(D)** Mean traces of PC SS after NE and Yoh application **(E)** Complex spike firing were neither further decreased, nor recovered after application of the α2-AR antagonist Yoh. **(F)** Complex spike CV2 values were not altered after Yoh application following NE injection. **(G)** Course of the mean complex spike firing of all PCs recorded shows that the firing frequency is not altered after application of NE and Yoh. Data are presented as mean±SEM. Statistical significance was evaluated by paired t-test. (*p≤0.05, **p≤0.01, ***p≤0.001).


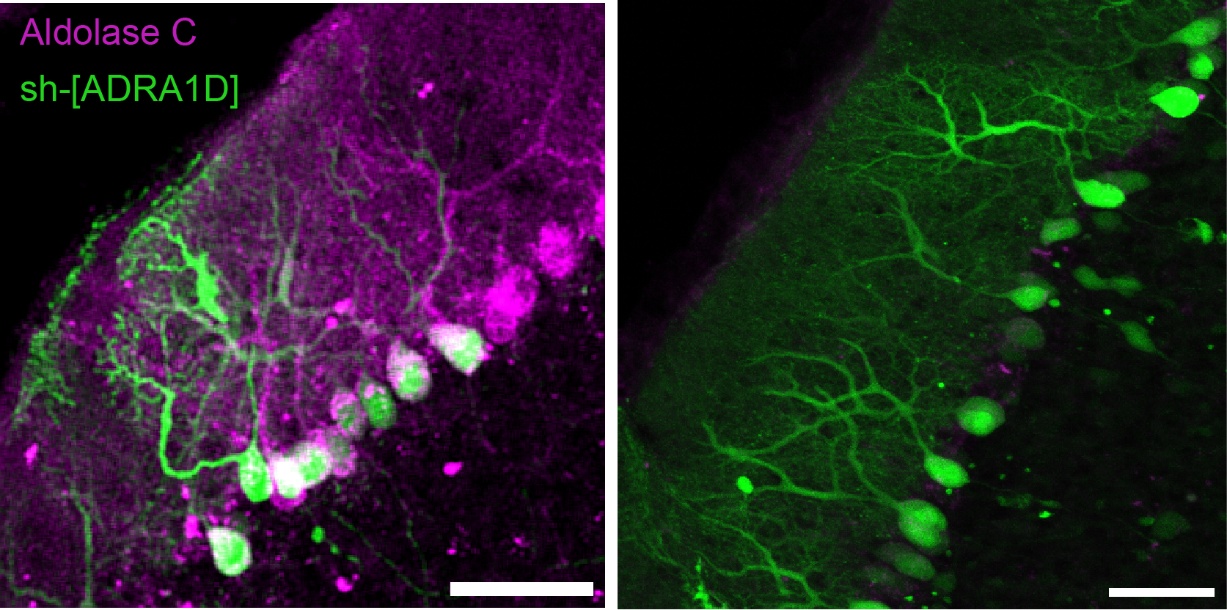


**Fig. S7: sh-[ADRA1D] (green) expresses in both aldolase C positive (magenta) and negative Purkinje cells.** Scale bars 50 µm.


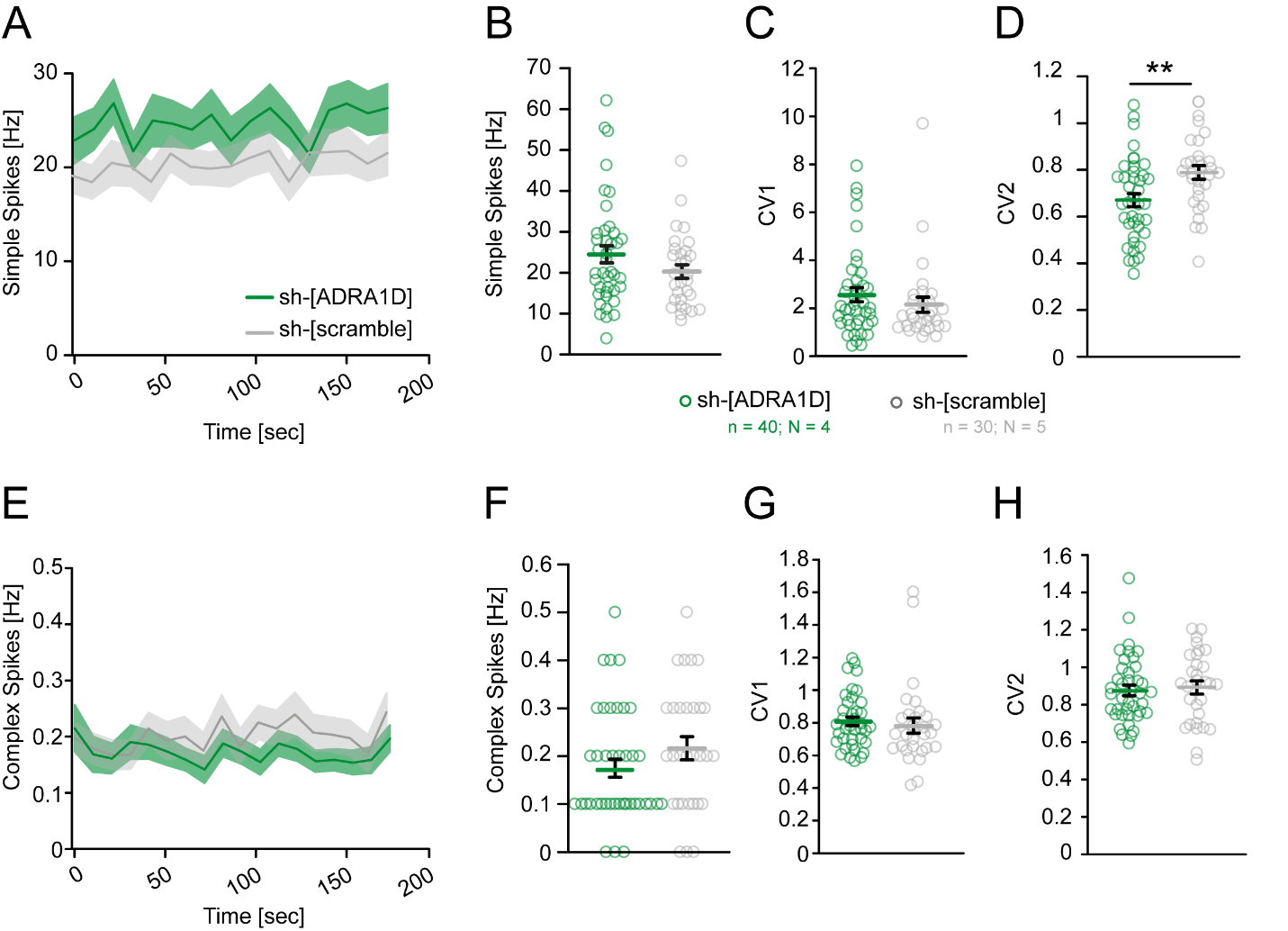
Fig. S8: Electrophysiological recordings reveal no differences in Purkinje cell simple spike and complex spike firing frequencies in sh-RNA injected tottering^tg/tg^ mice *in vivo*. (A) Mean firing frequencies Purkinje cell simple spikes of sh-[ADRA1D] (green, n=40, N=4) and sh-[scramble] (grey, n=30, N=3) injected tottering^tg/tg^ mice. Recordings were conducted for 180 sec. No significant differences were found in simple spike firing (B) or CV1 (C), but sh-[scramble] injected mice showed higher irregularities in their firing compared to sh-[ADRA1D] injected tottering^tg/tg^ mice (0.789±0.0294 vs 0.671±0.028, p=0.005) (D). (E) Corresponding mean complex spike traces of the recorded Purkinje cells shown in (A). No significant differences were found in the mean firing frequencies of complex spikes (F), CV1 (G) or CV2 (H) between the two groups. Data are presented as mean±SEM. Statistical significance was evaluated by paired t-test. (*p≤0.05, **p≤0.01, ***p≤0.001).

| **Test** | **Sample Size** | **Statistics** | **mean±SEM** | | **p-value** |
| --- | --- | --- | --- | --- | --- |
|  |  |  | NaCl | Prazosin |  |
| **Pole test** *time (s)* | n=10 | MW Rank Sum Test | 103.95±11.445 | 110.15±9.85 | p=0.584 |
| **Hang wire** *time (s)* | n=11 | Student’s t-test | 15.212±4.787 | 26.364±5.129 | p=0.128 |
| **Beam walk** |  |  |  |  |  |
| *time (s)* | n=10 | MW Rank Sum test | 112.933±7.067 | 102.033±9.898 | p=0.280 |
| *idle (s)* | n=10 | MW Rank Sum test | 0 | 2.167±1.138 | **p=0.035** |
| *slips right HP (n)* | n=10 | MW Rank Sum test | 0.667±0.667 | 2.15±1.247 | p=0.180 |
| *slips left HP (n)* | n=10 | MW Rank Sum test | 0.267±0.267 | 1.45±0.914 | p=0.280 |
| **Footprint Analysis** |  |  |  |  |  |
| *length right front paw (cm)* | n=11 | Student’s t-test | 6.602±0.178 | 6.685±0.188 | p=0.751 |
| *length left front paw (cm)* | n=11 | MW Rank Sum test | 6.605±0.154 | 6.636±0.16 | p=0.921 |
| *length right hind paw (cm)* | n=11 | Student’s t-test | 6.477±0.18 | 6.59±0.213 | p=0.883 |
| *length left hind paw (cm)* | n=11 | MW Rank Sum test | 6.155±0.284 | 6.319±0.287 | p=0.669 |
| *width front paws (cm)* | n=11 | MW Rank Sum test | 1.686±0.1223 | 1.685±0.135 | p=0.921 |
| *width hind paws (cm)* | n=11 | MW Rank Sum test | 3.126±0.179 | 3.165±0.167 | p=0.921 |

Table S1. Adrenoreceptor blocker prazosin hydrochloride has no impact on ataxia in tottering ^tg/tg^ mice. Significant p-values are highlighted in bold.

| **Test** | **Sample Size** | **Statistics** | **mean±SEM** | | **p-value** |
| --- | --- | --- | --- | --- | --- |
|  |  |  | NaCl | BMY-7378 |  |
| **Pole test** *time (s)* | n=10 | MW Rank Sum test | 76.955±10.20 | 34.385±9.089 | **p=0.031** |
| **Hang wire** *time (s)* | n=10 | Student’s t-test | 29.080±5.469 | 34.168±16.324 | p=0.507 |
| **Grip strength** |  |  |  |  |  |
| *grip force (g)* | n=10 | Student’s t-test | 59.550±7.999 | 71.440±7.183 | p=0.283 |
| **Footprint Analysis** |  |  |  |  |  |
| *length right front paw (cm)* | n=10 | Student’s t-test | 6.024±0.2061 | 5.180±0.1889 | **p=0.007** |
| *length left front paw (cm)* | n=10 | Student’s t-test | 5.76±0.2398 | 5.076±0.1723 | **p=0.033** |
| *length right hind paw (cm)* | n=10 | Student’s t-test | 6.03±0.2077 | 5.244±0.1142 | **p=0.004** |
| *length left hind paw (cm)* | n=10 | Student’s t-test | 6.006±0.21 | 5.194±0.1475 | **p=0.005** |
| *width front paws (cm)* | n=10 | Student’s t-test | 2.128±0.1371 | 2.338±0.1456 | p=0.307 |
| *width hind paws (cm)* | n=10 | Student’s t-test | 2.750±0.2142 | 3.288±0.2042 | p=0.086 |

Table S2. α1D-Adrenoreceptor blocker BMY-7378 dihydrochloride slightly improves ataxia in tottering ^tg/tg^ mice. Significant p-values are highlighted in bold.

| **Analysis** | **Sample Size** | **Statistics** | **mean±SEM**  **(Ref vs 2 mM BMY)** | **p-values** |
| --- | --- | --- | --- | --- |
| Simple Spikes [Hz] | N = 4; n = 16 | Wilcoxon Signed Rank Test | 34.525±2.926 vs 31.013±3.256 | p=0.002 |
| CV1 Simple Spikes |  | Wilcoxon Signed Rank Test | 0.618±0.0791 vs 0.702±0.0883 | p=0.159 |
| CV2 Simple Spikes |  | Wilcoxon Signed Rank Test | 0.504±0.0448 vs 0.541±0.0386 | p=0.175 |
| Complex Spikes (Hz) | N = 4; n = 16 | Paired t-test | 64.125±9.66 vs 56.438±7.674 | p=0.058 |
| CV1 Complex Spikes |  | Wilcoxon Signed Rank Test | 0.825±0.0389 vs 0.814±0.052 | p=0.464 |
| CV2 Complex Spikes |  | Paired t-test | 0.91±0.0441 vs 0838±0.0324 | p=0.129 |

Table S3. Pressure microinjection of 2mM BMY-7378 does impair PC SS firing

| Analysis | Sample Size | Statistics |  | mean±SEM | p-values |
| --- | --- | --- | --- | --- | --- |
| CV1 Simple Spikes Praz | n=12, N=4 | signed rank sum test | Ref vs NE | 2.123±0.22 vs 2.574±0.476 | p=0.85 |
|  |  | paired t-test | NE vs Praz | 2.574±0.476 vs 2.388±0.288 | p=0.741 |
|  |  | paired t-test | Ref vs Praz | 2.123±0.22 vs 2.388±0.288 | p=0.291 |
| CV1 Simple Spikes BMY-7378 | n=29, N=5 | signed rank sum test | Ref vs NE | 1.892±0.367 vs 2.449±0.512 | p=0.210 |
|  |  | signed rank sum test | NE vs BMY-7378 | 2.449±0.512 vs 1.786±0.363 | p=0.125 |
|  |  | signed rank sum test | Ref vs BMY-7378 | 1.892±0.367 vs 1.786±0.363 | p=1 |
| CV1 Simple Spikes Yoh | n=22, N=5 | signed rank sum test | Ref vs NE | 4.623±1.33 vs 3.229±2.516 | p=0.284 |
|  |  | signed rank sum test | NE vs Yoh | 3.229±2.516 vs 3.241±0.622 | p=0.194 |
|  |  | signed rank sum test | Ref vs Yoh | 4.623±1.33 vs 3.241±0.622 | **p=0.044** |
| CV1 Complex Spikes Praz | n=12, N=4 | Paired t-test | Ref vs NE | 0.564±0.0815 vs 0.753±0.0574 | p=0.059 |
|  |  | Paired t-test | NE vs Praz | 0.753±0.0574 vs 0.882±0.0619 | p=0.114 |
|  |  | Paired t-test | Ref vs Praz | 0.564±0.0815 vs 0.882±0.0619 | p=0.012 |
| CV1 Complex Spikes BMY-7378 | n=29, N=5 | Wilcoxon Signed Rank Test | Ref vs NE | 0.803±0.047 vs 0.951±0.0726 | p=0.333 |
|  |  | Paired t-test | NE vs BMY-7378 | 0.951±0.0726 vs 1.061±0.120 | p=0.192 |
|  |  | Wilcoxon Signed Rank Test | Ref vs BMY-7378 | 0.803±0.047 vs 1.061±0.120 | p=0.494 |
| CV1 Complex Spikes Yoh | n=22, N=5 | Paired t-test | Ref vs NE | 0.878±0.0565 vs 0.785±0.0483 | p=0.223 |
|  |  | Paired t-test | NE vs Yoh | 0.785±0.0483 vs 0.76±0.0564 | p=0.52 |
|  |  | Paired t-test | Ref vs Yoh | 0.878±0.0565 vs 0.76±0.0564 | p=0.082 |

Table S4. CV1 values of simple spikes and complex spikes after pressure injection of α1 and α2-AR antagonists.

| **Parameter** | **Sample Size** | **Statistics** |  | **mean±SEM** | **p-value** |
| --- | --- | --- | --- | --- | --- |
| ADRA1A | 9 vs 10 | Students t-test | Sh-[ADRA1D] vs sh-[scramble] | -1.302±0.144 vs 0±353 | p=0.004 |
| ADRA1B | 9 vs 10 | Students t-test | Sh-[ADRA1D] vs sh-[scramble] | -0.179±0.130 vs 0±0.200 | p=0.475 |
| ADRA2A | 9 vs 10 | MW Rank Sum Test | Sh-[ADRA1D] vs sh-[scramble] | -0.532±0.114 vs 0±0.0392 | p=0.001 |
| ADRA2B | 9 vs 10 | MW Rank Sum Test | Sh-[ADRA1D] vs sh-[scramble] | -0.749±0.334 vs 0±0.170 | p=0.066 |
| ADRA2C | 9 vs 10 | Students t-test | Sh-[ADRA1D] vs sh-[scramble] | -1.130±0.147 vs 0±0.287 | p=0.004 |

Table S5. ΔΔCT values of α1A, α1B, α2A, α2B and α2C after sh-[ADRA1D] and sh-[scramble] injection in tottering^tg/tg^ mice

| **Test** | **Sample Size n** | **Statistics** | **mean±SEM** | | **p-value** |
| --- | --- | --- | --- | --- | --- |
|  |  |  | **sh-[ADRA1D]** | **sh-[scramble]** |  |
| **Pole test** *time (s)* | 5 vs. 5 | MW Rank Sum Test | 66.778±14.381 | 94.492±18.119 | p=0.310 |
| **Hang wire** *time (s)* | 5 vs. 5 | Students t-test | 32.420±8.112 | 14.851±4.766 | p=0.099 |
| **Grip Strength** |  |  |  |  |  |
| *Grip force (g)* | 5 vs. 5 | Students t-test | 46.340±3.402 | 42.647±4.208 | p=0.514 |
| *force (f)* | 5 vs. 5 | Students t-test | 2.3±0.268 | 1.941±0.302 | p=0.40 |
| **Open Field test** |  |  |  |  |  |
| *Distance (cm)* | 5 vs. 5 | Students t-test | 3755.864±367.713 | 3338.382±217.417 | p=0.357 |
| *Velocity (cm/s)* | 5 vs. 5 | Students t-test | 4.198±0.410 | 3.735±0.236 | p=0.356 |
| *Duration at center (s)* | 5 vs. 5 | Students t-test | 78.719±29.408 | 56.266±12.876 | p=0.504 |
| *Duration at intermediate (s)* | 5 vs. 5 | Students t-test | 142.819±46.497 | 127.879±24.150 | p=0.783 |
| *Duration at border (s)* | 5 vs. 5 | MW Rank Sum Test | 677.653±75.677 | 713.620±27.246 | p=0.841 |
| **Footprint Analysis** |  |  |  |  |  |
| *length right front paw (cm)* | 5 vs. 5 | Students t-test | 58.640±5.454 | 56.920±9.364 | p=0.878 |
| *length left front paw (cm)* | 5 vs. 5 | Students t-test | 61.6±6.024 | 58.6±7.473 | p=0.763 |
| *length right hind paw (cm)* | 5 vs. 5 | Students t-test | 58.2±5.905 | 60.88±6.411 | p=0.766 |
| *length left hind paw (cm)* | 5 vs. 5 | Students t-test | 59.64±4.935 | 57.44±7.84 | p=0.818 |
| *width front paws (cm)* | 5 vs. 5 | Students t-test | 19.560±2.101 | 20.96±1.608 | p=0.611 |
| *width hind paws (cm)* | 5 vs. 5 | Students t-test | 36.08±1.089 | 38.72±1.256 | p=0.151 |

Table S6. Motor performance is not impaired after sh-[ADRA1D] or sh-[scramble] injected tottering^tg/tg^ mice. Significant p-values are highlighted in bold.

| **Primary Antibody** | **Dilution** | **Diluted in** | **supplier** |
| --- | --- | --- | --- |
| rb-α-α1_A_ | 1:400 | blocking buffer | Invitrogen^TM^, #PA1-047 |
| rb-α-α1_B_ | 1:200 | blocking buffer | alomone labs, #AAR-018 |
| rb-α-α1_D_ | 1:200 | blocking buffer | alomone labs, #AAR-019 |
| rb-α-α2_A_ | 1:200 | blocking buffer | Invitrogen^TM^, #PA1-048 |
| rb-α-α2_B_ | 1:200 | blocking buffer | alomone labs, #AAR-021 |
| rb-α-α2_C_ | 1:200 | blocking buffer | Invitrogen^TM^, #PA5-114828 |
| ms-α-Actin Ab-5 | 1:10000 | blocking buffer | BD Biosciences, #612656 |
| ms-α-Calbindin | 1:500 | blocking buffer | Sigma-Aldrich, C9848 |
| **Secondary Antibodies** |  |  |  |
| goat α-rb HRP-linked | 1:50000 | 1% milk powder, 0.5% BSA in 0.1% TBS-T | GE healthcare, NA934V5 |
| goat α-ms HRP-linked | 1:50000 | 1% milk powder, 0.5% BSA in 0.1% TBS-T | GE healthcare, NA931V5 |

Table S7. Primary and secondary antibodies used for western blot analysis.

| Primer name | Sequence |
| --- | --- |
| ADR α1_A_ fw | 5’- TGGCTGCCATTCTTCCTCGTGA -3’ |
| ADR α1_A_ rev | 5’- TTCTTGAACTCCTGGCTGGAGC -3’ |
| ADR α1_B_ fw | 5’- CCTTGGGCATTGTAGTCGGA -3’ |
| ADR α1_B_ rev | 5’- AAGTAGCCCAGCCAGAACAC -3’ |
| ADR α1_D_ fw | 5’- CTGCCAAGACCCTAGCCATC -3’ |
| ADR α1_D_ rev | 5’- GATGACCTTGAAGACGCCCT -3’ |
| ADR α2_A_ fw | 5’- CAGGTGACACTGACGCTGGTTT -3’ |
| ADR α2_A_ rev | 5’- GACACCAGGAAGAGGTTTTGGG -3’ |
| ADR α2_B_ fw | 5’- GTTCCAGCCTCGGCTAAAGT -3’ |
| ADR α2_B_ rev | 5’- GACCAATTGGGTGGCAAAGC -3’ |
| ADR α2_C_ fw | 5’- CTTGATCTGGGCCTCGACTG -3’ |
| ADR α2_C_ rev | 5’- GGTGCGCATCATTCCTTTGG -3’ |
| HPRT fw | 5’- AGTTCTTTGCTGACCTGCTG -3’ |
| HRPT rev | 5’- CCACCAATAACTTTTATGTCCCC -3’ |
| ACVRL1 fw | 5’- CCTAGTGCTATGGGAGATCGC -3’ |
| ACVRL1 rev | 5’- TGGGTGTCTGCTGGTCAAC -3’ |
| GAPDH fw | 5’- GCATTGTGGAAGGGCTCATG -3’ |
| GAPDH rev | 5’- TGCAGGGATGATGTTCTGGG -3’ |
| PGK1 fw | 5’- CCTGTTGACTTTGTCACTGC -3’ |
| PGK1 rev | 5’- CCCACAGCCTCGGCATATTT-3’ |
| CALB1 fw | 5’- ATTTCGACGCTGACGGAAGT -3’ |
| CALB1 rev | 5’- CCAATCCAGCCTTCTTTCGC -3’ |

Table S8. Used quantitative real-time PCR primers.

ACVRL1 = activin A receptor like type 1; ADR = adrenergic receptor; CALB1 = Calbindin 1, GDPDH = glyceraldehyde-3-phosphate dehydrogenase; HRPT = hypoxanthine guanine phosphoribosyl transferase; PGK1 = phosphoglycerate kinase 1.

| **Figure** | **Sample Size** | | **Statistics** |  | **Comparison** | | | **%±SEM** | **p-value** |
| --- | --- | --- | --- | --- | --- | --- | --- | --- | --- |
| 1A | n=11 | | MW Rank Sum Test | frequency | NaCl vs Praz | | | 72.0±0 vs 9.0±0 | **p=0.003** |
|  |  |  |  |  | NaCl vs Yoh | | | 72.0±0 vs 63.6±0 | p=0.684 |
|  |  |  |  |  | Praz vs Yoh | | | 9.0±0 vs 63.6±0 | **p=0.011** |
|  |  |  |  | | **comparison** | | | **mean±SEM** | **p-value** |
|  |  |  |  | onset (min) | NaCl vs Praz | | | 8.75±2.418 vs 10.0 | not tested |
|  |  |  | Students t-test |  | NaCl vs Yoh | | | 8.75±2.418 vs 5.286±1.426 | p=0.256 |
|  |  |  |  |  | Praz vs Yoh | | | 10.0 vs 5.286±1.426 | - |
|  |  |  |  | | **comparison** | | | **mean±SEM** | **p-value** |
|  |  |  |  | duration (min) | NaCl vs Praz | | | 37.875±7.11 vs 27.0 | - |
|  |  |  | Students t-test |  | NaCl vs Yoh | | | 37.875±7.11 vs 39.857±7.049 | p=0.847 |
|  |  |  |  |  | Praz vs Yoh | | | 27.0 vs 39.857±7.049 | - |
|  | | | | | **comparison** | | | **%±SEM** | **p-value** |
| 1B | n=5 | | MW Rank Sum Test | frequency | NaCl vs RS-17053 | | | 80.0 vs 80.0 | p=1.0 |
|  |  |  | Students t-test | onset (min) | NaCl vs RS-17053 | | | 7.5±3.304 vs 19.25±3.351 | **p=0.047** |
|  |  |  | Students t-test | duration (min) | NaCl vs RS-17053 | | | 74.75±12.195 vs 74.25±17.24 | p=0.982 |
|  | | | | | **comparison** | | | **mean±SEM** | **p-value** |
| 1C | n=15 | | MW Rank Sum Test | frequency | NaCl vs BMY-7378 | | | 66.67 vs 13.3 | **p=0.004** |
|  |  |  | Students t-test | onset (min) | NaCl vs BMY-7378 | | | 6.1±1.516 vs 7.5±6.5 | p=0.745 |
|  |  |  | Students t-test | duration (min) | NaCl vs BMY-7378 | | | 48.8±8.612 vs 33.0±7.0 | p=0.451 |
|  | | | | **Comparison** | | | | **time** | **p-value** |
| 1D | n=5 | | Two Way RM ANOVA  post-hoc Holm-Sidak | NaCl vs Praz | | | | -15 | p=1.0 |
|  |  |  |  |  |  |  |  | 0 | p=0.711 |
|  |  |  |  |  |  |  |  | 5 | p=0.276 |
|  |  |  |  |  |  |  |  | 10 | p=0.079 |
|  |  |  |  |  |  |  |  | 15 | **p=0.031** |
|  |  |  |  |  |  |  |  | 20 | **p=0.015** |
|  |  |  |  |  |  |  |  | 25 | **p=0.019** |
|  |  |  |  |  |  |  |  | 30 | **p=0.031** |
|  |  |  |  |  |  |  |  | 35 | **p=0.04** |
|  |  |  |  |  |  |  |  | 40 | **p=0.05** |
|  |  |  |  |  |  |  |  | 45 | p=0.099 |
|  |  |  |  |  |  |  |  | 50 | p=0.099 |
|  |  |  |  |  |  |  |  | 55 | **p=0.05** |
|  |  |  |  |  |  |  |  | 60 | p=0.079 |
|  |  |  |  |  |  |  |  | 65 | p=0.099 |
|  |  |  |  |  |  |  |  | 70 | p=0.187 |
|  |  |  |  |  |  |  |  | 75 | p=0.539 |
|  |  |  |  |  |  |  |  | 80 | p=0.462 |
|  |  |  |  |  |  |  |  | 85 | p=0.539 |
|  |  |  |  |  |  |  |  | 90 | p=0.805 |
|  |  |  |  |  |  |  |  | 95 | p=0.901 |
|  |  |  |  |  |  |  |  | 100 | p=1.0 |
|  |  | |  | **Comparison** | | | | **time** | **p-value** |
| 1D | N=5 | | Two Way RM ANOVA  post-hoc Holm-Sidak | NaCl vs BMY-7378 | | | | -15 | p=1.0 |
|  |  |  |  |  |  |  |  | 0 | p=0.516 |
|  |  |  |  |  |  |  |  | 5 | p=0.079 |
|  |  |  |  |  |  |  |  | 10 | p=0.366 |
|  |  |  |  |  |  |  |  | 15 | **p=0.021** |
|  |  |  |  |  |  |  |  | 20 | **p=0.012** |
|  |  |  |  |  |  |  |  | 25 | **p=0.019** |
|  |  |  |  |  |  |  |  | 30 | **p=0.021** |
|  |  |  |  |  |  |  |  | 35 | **p=0.036** |
|  |  |  |  |  |  |  |  | 40 | **p=0.047** |
|  |  |  |  |  |  |  |  | 45 | **p=0.028** |
|  |  |  |  |  |  |  |  | 50 | **p=0.047** |
|  |  |  |  |  |  |  |  | 55 | **p=0.036** |
|  |  |  |  |  |  |  |  | 60 | p=0.061 |
|  |  |  |  |  |  |  |  | 65 | p=0.079 |
|  |  |  |  |  |  |  |  | 70 | p=0.162 |
|  |  |  |  |  |  |  |  | 75 | p=0.516 |
|  |  |  |  |  |  |  |  | 80 | p=0.437 |
|  |  |  |  |  |  |  |  | 85 | p=0.516 |
|  |  |  |  |  |  |  |  | 90 | p=0.794 |
|  |  |  |  |  |  |  |  | 95 | p=0.896 |
|  |  |  |  |  |  |  |  | 100 | p=1.0 |
|  | **Sample size** | | **Statistics** | **Comparison** | | | | **%±SEM** | **p-value** |
| 1F | 10 vs 10 | | Students t-test | α1_A_ tottering^tg/tg^ vs tottering^-/-^ | | | | 5.031±0.254 vs.4.906±0.288 | p=0.564 |
|  |  | | MW Rank Sum Test | α1_B_ tottering^tg/tg^ vs tottering^-/-^ | | | | 7.805±0.866 vs.5.609±0.391 | **p=0.038** |
|  |  | | MW Rank Sum Test | α1_D_ tottering^tg/tg^ vs tottering^-/-^ | | | | 6.665±0.888 vs.4.273±0.434 | **p=0.045** |
|  |  | | Students t-test | α2_A_ tottering^tg/tg^ vs tottering^-/-^ | | | | 6.121±0.291 vs.5.894±0.298 | p=0.592 |
|  |  | | Students t-test | α2_B_ tottering^tg/tg^ vs tottering^-/-^ | | | | 8.841±0.225 vs.9.875±0.391 | **p=0.040** |
|  |  | | Students t-test | α2_C_ tottering^tg/tg^ vs tottering^-/-^ | | | | 6.923±0.288 vs.6.538±0.353 | p=0.410 |
| **Figure** | **Sample size** | | **statistics** | **comparison** | | | | **%±SEM** | **p-value** |
| 2C | n=12, N=4 | | paired t-test | Ref vs NE | | | | 23.325±4.281 vs 7.65±2.51 | **p≤0.001** |
|  |  |  | paired t-test | NE vs Praz | | | | 7.65±2.51 vs 18.942±3.209 | **p≤0.001** |
|  |  |  | paired t-test | Ref vs Praz | | | | 23.325±4.281 vs 18.942±3.209 | p=0.113 |
| 2D | n=12, N=4 | | paired t-test | Ref vs NE | | | | 0.866±0.0681 vs 0.977±0.0524 | p=0.114 |
|  |  |  | paired t-test | NE vs Praz | | | | 0.977±0.0524 vs 0.860±0.0657 | p=0.034 |
|  |  |  | paired t-test | Ref vs Praz | | | | 0.866±0.0681 vs 0.860±0.0657 | p=0.860 |
| 2F | n=29, N=5 | | paired t-test | Ref vs NE | | | | 30.203±1.94 vs 17.486±1.711 | **p≤0.001** |
|  |  |  | paired t-test | NE vs BMY-7378 | | | | 17.486±1.711 vs 22.031±1.77 | **p=0.013** |
|  |  |  | signed rank sum test | Ref vs BMY-7378 | | | | 30.203±1.94 vs  22.031±1.77 | **p≤0.001** |
| 2G | n=29, N=5 | | paired t-test | Ref vs NE | | | | 0.7±0.0249 vs 0.772±0.0268 | **p=0.002** |
|  |  |  | signed rank sum test | NE vs BMY-7378 | | | | 0.772±0.0268 vs 0.693±0.0362 | **p=0.015** |
|  |  |  | signed rank sum test | Ref vs BMY-7378 | | | | 0.7±0.0249 vs 0.693±0.0362 | p=0.067 |
| 2I | n=20, N=3 | | paired t-test | Ref vs BMY-7378 | | | | 19.88±1.712 vs 19.775±1.627 | p=0.818 |
|  |  |  | paired t-test | BMY-7378 vs NE | | | | 19.775±1.627 vs 17.3±1.574 | p=0.08 |
|  |  |  | paired t-test | Ref vs NE | | | | 19.88±1.712 vs 17.3±1.574 | p=0.054 |
| 2J | n=20, N=3 | | signed rank sum test | Ref vs BMY-7378 | | | | 0.663±0.044 vs 0.634±0.0429 | p=0.231 |
|  |  |  | paired t-test | BMY-7378 vs NE | | | | 0.634±0.0429 vs 0.657±0.0405 | p=0.320 |
|  |  |  | paired t-test | Ref vs NE | | | | 0.663±0.044 vs 0.657±0.0405 | p=0.831 |
| **Figure** | **Sample size** | | **statistics** | **comparison** | | | | **%±SEM** | **p-value** |
| 3D | 4 | | Student’s t-test | sucrose vs CNO | | | | 87.5±12.5 vs 83.3±16.667 | p=0.537 |
| 3E | 4 | | Student’s t-test | sucrose vs CNO | | | | 5.25±1.451 vs 8.5±0.518 | p=0.079 |
| 3F | 4 | | Student’s t-test | sucrose vs CNO | | | | 47.875±1.625 vs 40.875±7.503 | p=0.397 |
|  | **Sample size** | | **statistics** | **Comparison** | | | | **time** | **p-value** |
| 3G | 4 | | Two Way RM ANOVA  (p=0.029)  post-hoc Holm Sidak | sucrose vs CNO | | | | -15 | p=1 |
|  |  | |  |  | | | | 0 | p=0.813 |
|  |  | |  |  | | | | 5 | p=0.555 |
|  |  | |  |  | | | | 10 | p=0.009 |
|  |  | |  |  | | | | 15 | p=0.085 |
|  |  | |  |  | | | | 20 | p=0.032 |
|  |  | |  |  | | | | 25 | p=0.004 |
|  |  | |  |  | | | | 30 | p=0.053 |
|  |  | |  |  | | | | 35 | p=0.107 |
|  |  | |  |  | | | | 40 | p=0.723 |
|  |  | |  |  | | | | 45 | p=0.813 |
|  |  | |  |  | | | | 50 | p=0.813 |
|  |  | |  |  | | | | 55 | p=0.723 |
|  |  | |  |  | | | | 60 | p=0.723 |
|  |  | |  |  | | | | 65 | p=1 |
| **Figure** | **Sample size** | | **statistics** | **comparison** | | | | **%±SEM** | **p-value** |
| 4B | 5 vs 5 | | Two Way RM ANOVA  p≤0.001  post-hoc Holm-Sidak | frequency within ACSF  pre vs. 5d | | | | 93.33±6.667 vs 80.0 | p=0.749 |
|  |  |  |  | frequency within ACSF  pre vs. 7d | | | | 93.33±6.667 vs 80.0 | p=0.684 |
|  |  |  |  | frequency within ACSF  pre vs. 10d | | | | 93.33±6.667 vs 80.0 | p=0.842 |
|  |  |  |  | frequency within ACSF  pre vs. 14d | | | | 93.33±6.667 vs 100.0 | p=1.0 |
|  |  |  |  | frequency within ACSF  5d vs. 7d | | | | 80.0 vs 80.0 | p=1.0 |
|  |  |  |  | frequency within ACSF  5d vs. 10d | | | | 80.0 vs 80.0 | p=1.0 |
|  |  |  |  | frequency within ACSF  5d vs 14d | | | | 80.0 vs 100.0 | p=0.9 |
|  |  |  |  | frequency within ACSF  7d vs 10d | | | | 80.0 vs 80.0 | p=1.0 |
|  |  |  |  | frequency within ACSF  7d vs 14d | | | | 80.0 vs 100.0 | p=0.875 |
|  |  |  |  | frequency within ACSF  10d vs 14d | | | | 80.0 vs 100.0 | p=0.801 |
|  |  |  |  | frequency within BMY-7378  pre vs. 5d | | | | 86.667±6.667 vs 40.0 | **p=0.003** |
|  |  |  |  | frequency within BMY-7378  pre vs. 7d | | | | 86.667±6.667 vs 0.0 | **p≤0.001** |
|  |  |  |  | frequency within BMY-7378  pre vs. 10d | | | | 86.667±6.667 vs 0.0 | **p≤0.001** |
|  |  |  |  | frequency within BMY-7378  pre vs. 14d | | | | 86.667±6.667 vs. 0.0 | **p≤0.001** |
|  |  |  |  | frequency within BMY-7378  5d vs. 7d | | | | 40.0 vs 0.0 | p=0.084 |
|  |  |  |  | frequency within BMY-7378  5d vs. 10d | | | | 40.0 vs 0.0 | p=0.057 |
|  |  |  |  | frequency within BMY-7378  5d vs 14d | | | | 40.0 vs 0.0 | p=0.071 |
|  |  |  |  | frequency within BMY-7378  7d vs 10d | | | | 0.0 vs 0.0 | p=1.0 |
|  |  |  |  | frequency within BMY-7378  7d vs 14d | | | | 0.0 vs 0.0 | p=1.0 |
|  |  |  |  | frequency within BMY-7378  10d vs 14d | | | | 0.0 vs 0.0 | p=1.0 |
|  |  |  |  | frequency within pre  ACSF vs BMY-7378 | | | | 93.33±6.667 vs 86.667±6.667 | p=1.0 |
|  |  |  |  | frequency within 5d  ACSF vs BMY-7378 | | | | 80.0 vs 40.0 | **p=0.044** |
|  |  |  |  | frequency within 7d  ACSF vs BMY-7378 | | | | 80.0 vs 0.0 | **p≤0.001** |
|  |  |  |  | frequency within 10d  ACSF vs BMY-7378 | | | | 80.0 vs 0.0 | **p≤0.001** |
|  |  |  |  | frequency within 14d  ACSF vs BMY-7378 | | | | 100.0 vs 0.0 | **p≤0.001** |
|  | **Sample size** | | **statistics** | **comparison** | | | | **mean±SEM** | **p-value** |
| 4C | 5 | | Two Way RM ANOVA  p≤0.001  post-hoc Holm-Sidak | onset (min) within ACSF  pre vs. 5d | | | | 11.4±2.775 vs. 6.25±2.175 | p=0.987 |
|  |  |  |  | onset (min) within ACSF  pre vs. 7d | | | | 11.4±2.775 vs. 7.25±2.323 | p=0.989 |
|  |  |  |  | onset (min) within ACSF  pre vs. 10d | | | | 11.4±2.775 vs. 4.75±2.25 | p=0.884 |
|  |  |  |  | onset (min) within ACSF  pre vs. 14d | | | | 11.4±2.775 vs. 4.0±1.095 | p=0.435 |
|  |  |  |  | onset (min) within ACSF  5d vs. 7d | | | | 6.25±2.175 vs. 7.25±2.323 | p=0.976 |
|  |  |  |  | onset (min) within ACSF  5d vs. 10d | | | | 6.25±2.175 vs. 4.75±2.25 | p=0.988 |
|  |  |  |  | onset (min) within ACSF  5d vs 14d | | | | 6.25±2.175 vs. 4.0±1.095 | p=0.245 |
|  |  |  |  | onset (min) within ACSF  7d vs 10d | | | | 7.25±2.323 vs. 4.75±2.25 | p=0.993 |
|  |  |  |  | onset (min) within ACSF  7d vs 14d | | | | 7.25±2.323 vs. 4.0±1.095 | p=0.180 |
|  |  |  |  | 1 onset (min) within ACSF  0d vs 14d | | | | 4.75±2.25 vs. 4.0±1.095 | p=0.380 |
|  | 5 | | Two Way RM ANOVA  p≤0.001  post-hoc Holm-Sidak | onset (min) within BMY-7378  pre vs 5d | | | | 8.766±1.268 vs. 20.0±5.0 | **p≤0.001** |
|  |  |  |  | onset (min) within pre  ACSF vs BMY-7378 | | | | 11.4±2.775 vs. 8.766±1.268 | p=0.678 |
| 4D | 5 | | Two Way RM ANOVA  p≤0.001  post-hoc Holm-Sidak | duration (min) within ACSF  pre vs. 5d | | | | 57.365±4.809 vs. 45.25±5.851 | **p=0.003** |
|  |  |  |  | duration (min) within ACSF  pre vs. 7d | | | | 57.365±4.809 vs. 48.5±6.198 | **p=0.012** |
|  |  |  |  | duration (min) within ACSF  pre vs. 10d | | | | 57.365±4.809 vs. 44.75±5.558 | **p=0.003** |
|  |  |  |  | duration (min) within ACSF  pre vs. 14d | | | | 57.365±4.809 vs. 49.0±5.933 | p=0.417 |
|  |  |  |  | duration (min) within ACSF  5d vs. 7d | | | | 45.25±5.851 vs. 48.5±6.198 | p=0.862 |
|  |  |  |  | duration (min) within ACSF  5d vs. 10d | | | | 45.25±5.851 vs. 44.75±5.558 | p=0.941 |
|  |  |  |  | duration (min) within ACSF  5d vs. 14d | | | | 45.25±5.851 vs. 49.0±5.933 | p=0.126 |
|  |  |  |  | duration (min) within ACSF  7d vs. 10d | | | | 48.5±6.198 vs. 44.75±5.558 | p=0.924 |
|  |  |  |  | duration (min) within ACSF  7d vs. 14d | | | | 48.5±6.198 vs. 49.0±5.933 | p=0.284 |
|  |  |  |  | duration (min) within ACSF  10d vs.14d | | | | 44.75±5.558 vs. 49.0±5.933 | p=0.124 |
|  | 5 | | Two Way RM ANOVA  p≤0.001  post-hoc Holm-Sidak | duration (min) within BMY-7378  pre vs. 5d | | | | 55.632±5.593 vs. 12.5±6.5 | **p≤0.001** |
|  |  |  |  | duration (min) within BMY-7378  pre vs. 7d | | | | 55.632±5.593 vs. 0.0 | **p≤0.001** |
|  |  |  |  | duration (min) within BMY-7378  pre vs. 10d | | | | 55.632±5.593 vs. 0.0 | **p≤0.001** |
|  |  |  |  | duration (min) within BMY-7378  pre vs. 14d | | | | 55.632±5.593 vs. 0.0 | **p≤0.001** |
|  |  |  |  | duration (min) within BMY-7378  5d vs. 7d | | | | 12.5±6.5 vs. 0.0 | p=0.928 |
|  |  |  |  | duration (min) within BMY-7378  5d vs. 10d | | | | 12.5±6.5 vs. 0.0 | p=0.827 |
|  |  |  |  | duration (min) within BMY-7378  5d vs. 14d | | | | 12.5±6.5 vs. 0.0 | p=0.888 |
|  |  |  |  | duration (min) within BMY-7378  7d vs. 10d | | | | 0.0 vs. 0.0 | p=1.0 |
|  |  |  |  | duration (min) within BMY-7378  7d vs. 14d | | | | 0.0 vs. 0.0 | p=1.0 |
|  |  |  |  | duration (min) within BMY-7378  10d vs.14d | | | | 0.0 vs. 0.0 | p=1.0 |
|  |  |  |  | duration (min) within BMY-7378  17d (post 3d) | | | | 42.0±1.871 | - |
|  |  |  |  | duration (min) within BMY-7378  20d (post 7d) | | | | 37.2±1.655 | - |
|  |  |  |  | duration (min) within pre  ACSF vs BMY-7378 | | | | 57.365±4.809 vs. 55.632±5.593 | p=0.853 |
|  |  |  |  | duration (min) within 5d  ACSF vs BMY-7378 | | | | 45.25±5.851 vs. 12.5±6.5 | **p=0.004** |
|  |  |  |  | duration (min) within 7d  ACSF vs BMY-7378 | | | | 48.5±6.198 vs. 0.0 | **p≤0.001** |
|  |  |  |  | duration (min) within 10d  ACSF vs BMY-7378 | | | | 44.75±5.558 vs. 0.0 | **p=0.002** |
|  |  |  |  | duration (min) within 14d  ACSF vs BMY-7378 | | | | 49.0±5.933 vs. 0.0 | **p≤0.001** |
|  | **Sample size** | | **Statistics** | **Comparison** | | | | **time** | **p-value** |
| 4E | 5 vs 5 | | Two Way RM ANOVA  p≤0.001  post-hoc Holm-Sidack | 5d  ACSF vs BMY-7378 | | | | -15 | p=1 |
|  |  |  |  |  |  |  |  | 0 | p=1 |
|  |  |  |  |  |  |  |  | 5 | p=0.121 |
|  |  |  |  |  |  |  |  | 10 | **p=0.015** |
|  |  |  |  |  |  |  |  | 15 | **p≤0.001** |
|  |  |  |  |  |  |  |  | 20 | **p≤0.001** |
|  |  |  |  |  |  |  |  | 25 | **p≤0.001** |
|  |  |  |  |  |  |  |  | 30 | **p≤0.001** |
|  |  |  |  |  |  |  |  | 35 | **p=0.004** |
|  |  |  |  |  |  |  |  | 40 | **p=0.002** |
|  |  |  |  |  |  |  |  | 45 | **p=0.002** |
|  |  |  |  |  |  |  |  | 50 | p=0.340 |
|  |  |  |  |  |  |  |  | 55 | p=1 |
|  |  |  |  |  |  |  |  | 60 | p=1 |
| 4F | 5 vs 5 | | Two Way RM ANOVA  p≤0.001  post-hoc Holm-Sidack | 7d  ACSF vs BMY-7378 | | | | -15 | p=1 |
|  |  |  |  |  |  |  |  | 0 | p=0.218 |
|  |  |  |  |  |  |  |  | 5 | **p=0.017** |
|  |  |  |  |  |  |  |  | 10 | **p≤0.001** |
|  |  |  |  |  |  |  |  | 15 | **p≤0.001** |
|  |  |  |  |  |  |  |  | 20 | **p≤0.001** |
|  |  |  |  |  |  |  |  | 25 | **p≤0.001** |
|  |  |  |  |  |  |  |  | 30 | **p≤0.001** |
|  |  |  |  |  |  |  |  | 35 | **p≤0.001** |
|  |  |  |  |  |  |  |  | 40 | **p=0.002** |
|  |  |  |  |  |  |  |  | 45 | p=0.069 |
|  |  |  |  |  |  |  |  | 50 | p=0.535 |
|  |  |  |  |  |  |  |  | 55 | p=0.535 |
|  |  |  |  |  |  |  |  | 60 | p=0.535 |
| 4G | 5 vs 5 | | Two Way RM ANOVA  p≤0.001  post-hoc Holm-Sidack | 10d  ACSF vs BMY-7378 | | | | -15 | p=1 |
|  |  |  |  |  |  |  |  | 0 | p=0.124 |
|  |  |  |  |  |  |  |  | 5 | **p≤0.001** |
|  |  |  |  |  |  |  |  | 10 | **p≤0.001** |
|  |  |  |  |  |  |  |  | 15 | **p≤0.001** |
|  |  |  |  |  |  |  |  | 20 | **p≤0.001** |
|  |  |  |  |  |  |  |  | 25 | **p≤0.001** |
|  |  |  |  |  |  |  |  | 30 | **p≤0.001** |
|  |  |  |  |  |  |  |  | 35 | **p≤0.001** |
|  |  |  |  |  |  |  |  | 40 | **p=0.024** |
|  |  |  |  |  |  |  |  | 45 | p=0.057 |
|  |  |  |  |  |  |  |  | 50 | p=0.245 |
|  |  |  |  |  |  |  |  | 55 | p=0.436 |
|  |  |  |  |  |  |  |  | 60 | p=1 |
| 4H | 5 vs 5 | | Two Way RM ANOVA  p≤0.001  post-hoc Holm-Sidack | 14d  ACSF vs BMY-7378 | | | | -15 | p=1 |
|  |  |  |  |  |  |  |  | 0 | p=0.094 |
|  |  |  |  |  |  |  |  | 5 | **p≤0.001** |
|  |  |  |  |  |  |  |  | 10 | **p≤0.001** |
|  |  |  |  |  |  |  |  | 15 | **p≤0.001** |
|  |  |  |  |  |  |  |  | 20 | **p≤0.001** |
|  |  |  |  |  |  |  |  | 25 | **p≤0.001** |
|  |  |  |  |  |  |  |  | 30 | **p≤0.001** |
|  |  |  |  |  |  |  |  | 35 | **p≤0.001** |
|  |  |  |  |  |  |  |  | 40 | **p=0.001** |
|  |  |  |  |  |  |  |  | 45 | **p=0.018** |
|  |  |  |  |  |  |  |  | 50 | p=0.094 |
|  |  |  |  |  |  |  |  | 55 | p=0.467 |
|  |  |  |  |  |  |  |  | 60 | p=0.467 |
|  |  |  |  |  |  |  |  | 65 | p=0.808 |
| **Figure** | **Sample size** | | **statistics** | **Comparison** | | | | **%±SEM** | **p-value** |
| 5C | 10 | | Two Way RM ANOVA  p≤0.001  post-hoc Holm-Sidack | frequency within shRNA pre vs 3 weeks | | | | 80.0±10.0 vs 20.0±0.0 | **p≤0.001** |
|  | 10 | |  | frequency within shRNA pre vs 4 weeks | | | | 80.0±10.vs 0.0 | **p≤0.001** |
|  | 10 | |  | frequency within shRNA pre vs 5 weeks | | | | 80.0±10.vs 0.0 | **p≤0.001** |
|  | 10 | |  | frequency within shRNA pre vs 6 weeks | | | | 80.0±10.vs 0.0 | **p≤0.001** |
|  | 10 | |  | frequency within shRNA 3weeks vs 4 weeks | | | | 20.0±0.0 vs 0.0 | **p=0.037** |
|  | 10 | |  | frequency within shRNA 3weeks vs 5 weeks | | | | 20.0±0.0 vs 0.0 | **p=0.031** |
|  | 10 | |  | frequency within shRNA 3weeks vs 6 weeks | | | | 20.0±0.0 vs 0.0 | **p=0.025** |
|  | 10 | |  | frequency within shRNA 4 weeks vs 5 weeks | | | | 0.0 vs 0.0 | p=1 |
|  | 10 | |  | frequency within shRNA 4 weeks vs 6 weeks | | | | 0.0 vs 0.0 | p=1 |
|  | 10 | |  | frequency within shRNA 5 weeks vs 6 weeks | | | | 0.0 vs 0.0 | p=1 |
|  | 5 | |  | frequency within scrambleRNA pre vs 3 weeks | | | | 86.667±6.667 vs 100 | p=1 |
|  | 5 | |  | frequency within scrambleRNA pre vs 4 weeks | | | | 86.667±6.667 vs 100 | p=1 |
|  | 5 | |  | frequency within scrambleRNA pre vs 5 weeks | | | | 86.667±6.667 vs 100 | p=1 |
|  | 5 | |  | frequency within scrambleRNA pre vs 6 weeks | | | | 86.667±6.667 vs 100 | p=1 |
|  | 5 | |  | frequency within scrambleRNA 3 weeks vs 4 weeks | | | | 100 vs 100 | p=1 |
|  | 5 | |  | frequency within scrambleRNA 3 weeks vs 5 weeks | | | | 100 vs 100 | p=1 |
|  | 5 | |  | frequency within scrambleRNA 3 weeks vs 6 weeks | | | | 100 vs 100 | p=1 |
|  | 5 | |  | frequency within scrambleRNA 4 weeks vs 5 weeks | | | | 100 vs 100 | p=1 |
|  | 5 | |  | frequency within scrambleRNA 4 weeks vs 6 weeks | | | | 100 vs 100 | p=1 |
|  | 5 | |  | frequency within scrambleRNA 5 weeks vs 6 weeks | | | | 100 vs 100 | p=1 |
|  | 10 vs 5 | |  | frequency within pre | | | | 80.0±10.0 vs 86.667±6.667 | p=1 |
|  | 10 vs 5 | |  | frequency within 3 weeks | | | | 20.0±0.0 vs 100 | **p≤0.001** |
|  | 10 vs 5 | |  | frequency within 4 weeks | | | | 0 vs 100 | **p≤0.001** |
|  | 10 vs 5 | |  | frequency within 5 weeks | | | | 0 vs 100 | **p≤0.001** |
|  | 10 vs 5 | |  | frequency within 6 weeks | | | | 0 vs 100 | **p≤0.001** |
|  | **Sample size** | | **statistics** | **Comparison** | | | | **mean±SEM** | **p-value** |
| 5D | 10 vs 5 | | Two Way RM ANOVA  p=0.838  post-hoc Holm-Sidack | not tested | | | |  |  |
| 5E | 10 | | Two Way RM ANOVA  p=0.019  post-hoc Holm-Sidack | duration (min) within shRNA pre vs 3 weeks | | | | 67.816±7.782 vs 40.0±17 | **p≤0.001** |
|  | 10 | |  | duration (min) within shRNA pre vs 4 weeks | | | | 67.816±7.782 vs 0.0 | **p≤0.001** |
|  | 10 | |  | duration (min) within shRNA pre vs 5 weeks | | | | 67.816±7.782 vs 0.0 | **p≤0.001** |
|  | 10 | |  | duration (min) within shRNA pre vs 6 weeks | | | | 67.816±7.782 vs 0.0 | **p≤0.001** |
|  | 10 | |  | duration (min) within shRNA 3weeks vs 4 weeks | | | | 40.0±17 vs 0.0 | p=0.907 |
|  | 10 | |  | duration (min) within shRNA 3weeks vs 5 weeks | | | | 40.0±17 vs 0.0 | p=0.942 |
|  | 10 | |  | duration (min) within shRNA 3weeks vs 6 weeks | | | | 40.0±17 vs 0.0 | p=0.851 |
|  | 10 | |  | duration (min) within shRNA 4 weeks vs 5 weeks | | | | 0.0 vs 0.0 | p=1 |
|  | 10 | |  | duration (min) within shRNA 4 weeks vs 6 weeks | | | | 0.0 vs 0.0 | p=1 |
|  | 10 | |  | duration (min) within shRNA 5 weeks vs 6 weeks | | | | 0.0 vs 0.0 | p=1 |
|  | 5 | |  | duration (min) within scrambleRNA pre vs 3 weeks | | | | 86.032±17.131 vs 79.4±20.825 | p=0.844 |
|  | 5 | |  | duration (min) within scrambleRNA pre vs 4 weeks | | | | 86.032±17.131 vs 49.6±5.316 | p=0.059 |
|  | 5 | |  | duration (min) within scrambleRNA pre vs 5 weeks | | | | 86.032±17.131 vs 52.0±6.465 | p=0.087 |
|  | 5 | |  | duration (min) within scrambleRNA pre vs 6 weeks | | | | 86.032±17.131 vs 15.255±6.822 | p=0.254 |
|  | 5 | |  | duration (min) within scrambleRNA 3 weeks vs 4 weeks | | | | 79.4±20.825 vs 49.6±5.316 | p=0.171 |
|  | 5 | |  | duration (min) within scrambleRNA 3 weeks vs 5 weeks | | | | 79.4±20.825 vs 52.0±6.465 | p=0.227 |
|  | 5 | |  | duration (min) within scrambleRNA 3 weeks vs 6 weeks | | | | 79.4±20.825 vs 15.255±6.822 | p=0.524 |
|  | 5 | |  | duration (min) within scrambleRNA 4 weeks vs 5 weeks | | | | 49.6±5.316 vs 52.0±6.465 | p=0.851 |
|  | 5 | |  | duration (min) within scrambleRNA 4 weeks vs 6 weeks | | | | 49.6±5.316 vs 15.255±6.822 | p=0.878 |
|  | 5 | |  | duration (min) within scrambleRNA 5 weeks vs 6 weeks | | | | 52.0±6.465 vs 15.255±6.822 | p=0.891 |
|  | 10 vs 5 | |  | duration (min) within pre | | | | 67.816±7.782 vs 86.032±17.131 | p=0.98 |
|  | 10 vs 5 | |  | duration (min) within 3 weeks | | | | 40.0±17 vs 79.4±20.825 | **p≤0.001** |
|  | 10 vs 5 | |  | duration (min) within 4 weeks | | | | 0.0 vs 49.6±5.316 | **p≤0.001** |
|  | 10 vs 5 | |  | duration (min) within 5 weeks | | | | 0.0 vs 52.0±6.465 | **p≤0.001** |
|  | 10 vs 5 | |  | duration (min) within 6 weeks | | | | 0.0 vs 15.255±6.822 | **p≤0.001** |
|  | **Sample size** | | **statistics** | **Comparison** | | | | **time** | **p-value** |
| 5F | 10 vs 5 | | Two Way RM ANOVA  p≤0.001  post-hoc Holm-Sidack | week 3 post injection  shRNA vs scrambleRNA | | | | -15 | p=1 |
|  |  |  |  |  |  |  |  | 0 | **p≤0.001** |
|  |  |  |  |  |  |  |  | 5 | **p≤0.001** |
|  |  |  |  |  |  |  |  | 10 | **p≤0.001** |
|  |  |  |  |  |  |  |  | 15 | **p≤0.001** |
|  |  |  |  |  |  |  |  | 20 | **p≤0.001** |
|  |  |  |  |  |  |  |  | 25 | **p≤0.001** |
|  |  |  |  |  |  |  |  | 30 | **p≤0.001** |
|  |  |  |  |  |  |  |  | 35 | **p≤0.001** |
|  |  |  |  |  |  |  |  | 40 | **p≤0.001** |
|  |  |  |  |  |  |  |  | 45 | **p≤0.001** |
|  |  |  |  |  |  |  |  | 50 | **p≤0.001** |
|  |  |  |  |  |  |  |  | 55 | **p=0.002** |
|  |  |  |  |  |  |  |  | 60 | **p=0.009** |
|  |  |  |  |  |  |  |  | 65 | **p=0.009** |
|  |  |  |  |  |  |  |  | 70 | **p=0.009** |
|  |  |  |  |  |  |  |  | 75 | **p=0.009** |
|  |  |  |  |  |  |  |  | 80 | **p=0.009** |
|  |  |  |  |  |  |  |  | 85 | p=0.097 |
|  |  |  |  |  |  |  |  | 90 | p=0.315 |
|  |  |  |  |  |  |  |  | 95 | p=0.182 |
|  |  |  |  |  |  |  |  | 100 | **p=0.047** |
|  |  |  |  |  |  |  |  | 105 | p=0.097 |
|  |  |  |  |  |  |  |  | 110 | p=0.182 |
|  |  |  |  |  |  |  |  | 115 | p=0.182 |
|  |  |  |  |  |  |  |  | 120 | p=1 |
|  |  |  |  |  |  |  |  | 125 | p=0.182 |
|  | **Sample size** | | **statistics** | **Comparison** | | | | **mean±SEM** | **p-value** |
| 5G | 14 vs 15 | | Student’s t-test | sh-[ADRA1D] vs sh-[scramble] | | | | 0.287±0.0665 vs 0.738±0.108 | **p=0.035** |
| 5H | **10 vs 15** | | Mann-Whitney Rank Sum Test | sh-[ADRA1D] vs sh-[scramble] | | | | 0.277±0.0734 vs 0.738±0.183 | **p=0.038** |
|  | 10 vs 5 | | Student’s t-test | sh-[ADRA1D] vs tottering^tg/tg^ | | | | 0.277±0.0734 vs 0.510±0.0703 | p=0.066 |
|  | 15 vs 5 | | Mann-Whitney Rank Sum Test | sh-[scramble] vs tottering^-/-^ | | | | 0.738±0.183 vs 0.905±0.160 | p=0.205 |
|  | 15 vs 7 | | Mann-Whitney Rank Sum Test | sh-[scramble] vs tottering^tg/tg^ | | | | 0.738±0.183 vs 0.510±0.0703 | p=0.727 |
|  | 5 vs 7 | | Student’s t-test | tottering^-/-^ vs tottering^tg/tg^ | | | | 0.905±0.160 vs 0.510±0.0703 | p=0.077 |
| 5I | **Sample size** | | **statistics** | **Comparison** | | | | **mean±SEM** | **p-value** |
|  | n = 3;  N = 22 | | Mann-Whitney Rank Sum Test | sh-[ADRA1D] Simple Spikes | | | Pre vs. post NE | 18.932±1.684 vs 17.545±1.569 | p=0.614 |
|  |  |  | Student’s t-test | sh-[ADRA1D] Simple Spikes CV2 | | | Pre vs. post NE | 0.652±0.0383 vs 0.642±0.0313 | p=0.838 |
| 5J | **Sample size** | | **statistics** | **Comparison** | | | | **mean±SEM** | **p-value** |
|  | n = 5;  N = 32 | | Mann-Whitney Rank Sum Test | sh-[scramble] Simple Spikes | | | Pre vs. post NE | 19.837±9.764 vs 12.222±2.052 | **p=0.001** |
|  |  |  | Student’s t-test | sh-[scramble] Simple Spikes CV2 | | | Pre vs. post NE | 0.778±0.0305 vs 0.856±0.0347 | p =0.098 |
| **Figure** | **Sample size** | | **statistics** | **Comparison** | | | | **mean±SEM** | **p-value** |
| 6E | | 204 NaCl vs 205 BMY | Wilcoxon Signed Rank Test | ROI activity | | NaCl pre vs during | | 3.843±0.0639 vs 3.901±0.0716 | p=0.317 |
|  |  |  | Paired t-test |  |  | NaCl pre vs post | | 3.843±0.0639 vs 3.877±0.0616 | p=0.587 |
|  |  |  | Wilcoxon Signed Rank Test |  |  | NaCl during vs post | | 3.901±0.0716 vs 3.877±0.0616 | p=0.706 |
|  |  |  | Wilcoxon Signed Rank Test |  |  | BMY pre vs during | | 3.222±0.0653 vs 3.039±0.0666 | **p≤0.001** |
|  |  |  | Wilcoxon Signed Rank Test |  |  | BMY pre vs post | | 3.222±0.0653 vs 3.097±0.668 | p=0.211 |
|  |  |  | Wilcoxon Signed Rank Test |  |  | BMY during vs post | | 3.097±0.668vs 3.097±0.668 | p=0. 191 |
|  |  |  | MW Rank Sum test |  |  | pre NaCl vs BMY | | 3.843±0.0639 vs 3.222±0.0653 | **p≤0.001** |
|  |  |  | Student’s t-test |  |  | during NaCl vs BMY | | 3.901±0.0716 vs 3.097±0.668 | **p≤0.001** |
|  |  |  | Student’s t-test |  |  | post NaCl vs BMY | | 3.877±0.0616 vs 3.097±0.668 | **p≤0.001** |
| 6G | |  | Wilcoxon Signed Rank Test | ΔF/F | | NaCl pre vs during | | 0.489±0.0315 vs 0.586±0.0453 | **p≤0.001** |
|  |  |  | Wilcoxon Signed Rank Test |  |  | NaCl pre vs post | | 0.489±0.0315 vs 0.515±0.0465 | p=0.085 |
|  |  |  | Wilcoxon Signed Rank Test |  |  | NaCl during vs post | | 0.586±0.0453 vs 0.515±0.0465 | **p≤0.001** |
|  |  |  | Wilcoxon Signed Rank Test |  |  | BMY pre vs during | | 0.336±0.0231 vs 0.261±0.0159 | **p≤0.001** |
|  |  |  | Wilcoxon Signed Rank Test |  |  | BMY pre vs post | | 0.336±0.0231 vs 0.228±0.0147 | **p≤0.001** |
|  |  |  | Wilcoxon Signed Rank Test |  |  | BMY during vs post | | 0.261±0.0159 vs 0.228±0.0147 | p=0.091 |
|  |  |  | MW Rank Sum test |  |  | pre NaCl vs BMY | | 0.489±0.0315 vs 0.336±0.0231 | **p≤0.001** |
|  |  |  | MW Rank Sum test |  |  | during NaCl vs BMY | | 0.586±0.0453 vs 0.261±0.0159 | **p≤0.001** |
|  |  |  | MW Rank Sum test |  |  | post NaCl vs BMY | | 0.515±0.0465 vs 0.228±0.0147 | **p≤0.001** |
| 6I | 204 NaCl vs 205 BMY | | Wilcoxon Signed Rank Test | Peak Height | | NaCl pre vs during | | 0.0133±0.000698 vs 0.0156±0.000994 | **p=0.004** |
|  |  |  | Wilcoxon Signed Rank Test |  |  | NaCl pre vs post | | 0.0133±0.000698 vs 0.013±0.000912 | **p=0.003** |
|  |  |  | Wilcoxon Signed Rank Test |  |  | NaCl during vs post | | 0.0156±0.000994 vs 0.013±0.000912 | **p≤0.001** |
|  |  |  | Wilcoxon Signed Rank Test |  |  | BMY pre vs during | | 0.011±0.000497 vs 0.00957±0.000369 | **p≤0.001** |
|  |  |  | Wilcoxon Signed Rank Test |  |  | BMY pre vs post | | 0.011±0.000497 vs 0.00888±0.000359 | **p≤0.001** |
|  |  |  | Wilcoxon Signed Rank Test |  |  | BMY during vs post | | 0.00957±0.000369 vs 0.00888±0.000359 | **p=0.012** |
|  |  |  | MW Rank Sum test |  |  | pre NaCl vs BMY | | 0.0133±0.000698 vs 0.011±0.000497 | **p≤0.001** |
|  |  |  | MW Rank Sum test |  |  | during NaCl vs BMY | | 0.0156±0.000994 vs 0.00957±0.000369 | **p≤0.001** |
|  |  |  | MW Rank Sum test |  |  | post NaCl vs BMY | | 0.013±0.000912 vs 0.00888±0.000359 | p=0.173 |
| 6J | 204 NaCl vs 205 BMY | | Mann-Whitney Rank Sum Test | CV1 | | NaCl pre vs during | | 0.982±0.0193 vs 0.965±0.0209 | p=0.315 |
|  |  |  | Mann-Whitney Rank Sum Test |  |  | NaCl pre vs post | | 0.982±0.0193 vs 1.059±0.0234 | **p=0.028** |
|  |  |  | Mann-Whitney Rank Sum Test |  |  | NaCl during vs post | | 0.965±0.0209 vs 1.059±0.0234 | **p=0.003** |
|  |  |  | Mann-Whitney Rank Sum Test |  |  | BMY pre vs during | | 1.216±0.0214 vs 1.292±0.0217 | **p=0.005** |
|  |  |  | Mann-Whitney Rank Sum Test |  |  | BMY pre vs post | | 1.216±0.0214 vs 1.313±0.0197 | **p≤0.001** |
|  |  |  | Mann-Whitney Rank Sum Test |  |  | BMY during vs post | | 1.292±0.0217 vs 1.313±0.0197 | p=0.465 |
|  |  |  | Mann-Whitney Rank Sum Test |  |  | pre NaCl vs BMY | | 0.982±0.0193 vs 1.216±0.0214 | **p≤0.001** |
|  |  |  | Mann-Whitney Rank Sum Test |  |  | during NaCl vs BMY | | 0.965±0.0209 vs 1.292±0.0217 | **p≤0.001** |
|  |  |  | Mann-Whitney Rank Sum Test |  |  | post NaCl vs BMY | | 1.059±0.0234 vs 1.313±0.0197 | **p≤0.001** |
| 6K | 204 NaCl vs 205 BMY | | Mann-Whitney Rank Sum Test | CV2 | | NaCl pre vs during | | 0.266±0.00674 vs 0.232±0.00696 | **p≤0.001** |
|  |  |  | Mann-Whitney Rank Sum Test |  |  | NaCl pre vs post | | 0.266±0.00674 vs 0.262±0.00797 | p=0.424 |
|  |  |  | Mann-Whitney Rank Sum Test |  |  | NaCl during vs post | | 0.232±0.00696 vs 0.262±0.00797 | **p=0.015** |
|  |  |  | Mann-Whitney Rank Sum Test |  |  | BMY pre vs during | | 0.297±0.00537 vs 0.340±0.00513 | **p≤0.001** |
|  |  |  | Mann-Whitney Rank Sum Test |  |  | BMY pre vs post | | 0.297±0.00537 vs 0.351±0.00542 | **p≤0.001** |
|  |  |  | Mann-Whitney Rank Sum Test |  |  | BMY during vs post | | 0.340±0.00513 vs 0.351±0.00542 | p=0.183 |
|  |  |  | Mann-Whitney Rank Sum Test |  |  | pre NaCl vs BMY | | 0.266±0.00674 vs 0.297±0.00537 | **p≤0.001** |
|  |  |  | Mann-Whitney Rank Sum Test |  |  | during NaCl vs BMY | | 0.232±0.00696 vs 0.340±0.00513 | **p≤0.001** |
|  |  |  | Mann-Whitney Rank Sum Test |  |  | post NaCl vs BMY | | 0.262±0.00797 vs 0.351±0.00542 | **p≤0.001** |

Table S9. List of statistical tests, p-values and data. Significant p-values are highlighted in bold.

|  | **Sample Size** | **Statistics** | **Comparison** | **mean±SEM** | **p-values** |
| --- | --- | --- | --- | --- | --- |
| S3B | n=44, N=6 tottering^-/-^  n=33, N=4 tottering^tg/tg^ | MW Rank Sum test | Simple Spikes | 30.289±1.657 vs 26.43±2.778 | p=0.142 |
|  |  | MW Rank Sum test | CV1 | 0.71±0.0769 vs 1.765±0.216 | **p≤0.001** |
|  |  | MW Rank Sum test | CV2 | 0.470±0.0179 vs 0.728±0.0285 | **p≤0.001** |
| S3C | n=44, N=6 tottering^-/-^  n=33, N=4 tottering^tg/tg^ | MW Rank Sum test | Complex Spikes | 0.232±0.0245 vs 0.145±0.0169 | **p=0.012** |
|  |  | MW Rank Sum test | CV1 | 0.855±0.0349 vs 0.895±0.0406 | p=0.53 |
|  |  | Student’s t-test | CV2 | 0.857±0.015 vs 0.901±0.0233 | p=0.108 |
| S4C | N = 3;  n = 34 | Paired t-test | Simple Spikes  Reference vs NE | 30.146±1.697 vs 16.340±1.996 | **p≤0.001** |
|  | N = 3;  n = 34 | Wilcoxon Signed Rank Test | CV1  Reference vs NE | 1.356±0.218 vs 3.261±1.085 | p=0.242 |
|  | N = 3;  n = 34 | Wilcoxon Signed Rank Test | CV2  Reference vs NE | 0.632±0.0242 vs 0.732±0.0303 | **p≤0.001** |
|  | N = 3;  n = 34 | Wilcoxon Signed Rank Test | Complex Spikes  Reference vs NE | 0.179±0.0183 vs 0.103±0.0171 | **p=0.003** |
|  | N = 3;  n = 34 | Paired t-test | CV1  Reference vs NE | 0.832±0.0304 vs 0.881±0.0681 | p=0.335 |
|  | N = 3;  n = 34 | Wilcoxon Signed Rank Test | CV2  Reference vs NE | 0.914±0.0305 vs 0.927±0.0418 | p=0.459 |
| S5A | n=12, N=4 | Paired t-test | Ref vs NE | 0.3±0.127 vs 0.338±0.128 | p=1 |
|  |  | Wilcoxon Signed Rank Test | NE vs Praz | 0.338±0.128 vs | p=0.688 |
|  |  | Paired t-test | Ref vs Praz | 0.3±0.127 vs 0.35±0.117 | p=0.594 |
| S5B | n=12, N=4 | Paired t-test | Ref vs NE | 0.736±0.0731 vs 0.9±0.0679 | p=0.086 |
|  |  | Paired t-test | NE vs Praz | 0.9±0.0679 vs 0.931±0.0672 | p=0.956 |
|  |  | Paired t-test | Ref vs Praz | 0.736±0.0731 vs 0.931±0.0672 | p=0.201 |
| S5D | n=29, N=5 | Wilcoxon Signed Rank Test | Ref vs NE | 0.248±0.037 vs 0.189±0.033 | **p=0.007** |
|  |  | Wilcoxon Signed Rank Test | NE vs BMY-7378 | 0.189±0.033 vs 0.192±0.0312 | p=0.104 |
|  |  | Wilcoxon Signed Rank Test | Ref vs BMY-7378 | 0.248±0.037 vs 0.192±0.0312 | **p=0.003** |
| S5E | n=29, N=5 | Paired t-test | Ref vs NE | 0.930±0.03 vs 0.938±0.0426 | p=0.877 |
|  |  | Paired t-test | NE vs BMY-7378 | 0.938±0.0426 vs 0.848±0.0423 | p=0.482 |
|  |  | Paired t-test | Ref vs BMY-7378 | 0.930±0.03 vs ± 0.848±0.0423 | p=0.005 |
| S5G | n=20, N=3 | Wilcoxon Signed Rank Test | Ref vs BMY-7378 | 0.115±0.0296 vs 0.108±0.0309 | p=0.461 |
|  |  | Wilcoxon Signed Rank Test | BMY-7378 vs NE | 0.108±0.0309 vs 0.0563±0.0203 | p=0.063 |
|  |  | Paired t-test | Ref vs NE | 0.115±0.0296 vs 0.0563±0.0203 | p=0.082 |
| S5H | n=20, N=3 | MW Rank Sum test | Ref vs BMY-7378 | 0.984±0.0952 vs 1.059±0.196 | p=0.847 |
|  |  | MW Rank Sum test | BMY-7378 vs NE | 1.059±0.196 vs 0.784±0.0846 | p=0.512 |
|  |  | Student’s t-test | Ref vs NE | 0.984±0.0952 vs 0.784±0.0846 | p=0.132 |
| S6B | n=22, N=5 | signed rank sum test | Ref vs NE | 25.014±2.318 vs 14.882±1.657 | **p≤0.001** |
|  |  | signed rank sum test | NE vs Yoh | 14.882±1.657 vs 13.464±1.642 | p=0.055 |
|  |  | paired t-test | Ref vs Yoh | 25.014±2.318 vs 13.464±1.642 | **p≤0.001** |
| S6C |  | paired t-test | Ref vs NE | 0.788±0.0468 vs 0.828±0.0304 | p=0.195 |
|  |  | signed rank sum test | NE vs Yoh | 0.828±0.0304 vs 0.868±0.0322 | p=0.098 |
|  |  | paired t-test | Ref vs Yoh | 0.788±0.0468 vs 0.868±0.0322 | **p=0.006** |
| S6E |  | Wilcoxon Signed Rank Test | Ref vs NE | 0.162±0.0253 vs 0.14±0.0184 | p=0.542 |
|  |  | Wilcoxon Signed Rank Test | NE vs Yoh | 0.14±0.0184 vs 0.121±0.0196 | p=0.175 |
|  |  | Wilcoxon Signed Rank Test | Ref vs Yoh | 0.162±0.0253 vs 0.121±0.0196 | p=0.083 |
| S6F |  | Paired t-test | Ref vs NE | 0.942±0.0428 vs 0.902±0.0529 | p=0.724 |
|  |  | Paired t-test | NE vs Yoh | 0.902±0.0529 vs 0.878±0.0493 | p=0.680 |
|  |  | Paired t-test | Ref vs Yoh | 0.942±0.0428 vs 0.878±0.0493 | p=0.301 |
|  | **Sample Size** | **Statistics** | **Comparison** | **mean±SEM** | **p-values** |
| S7B | n=40; N=4 sh-[ADRA1D)] vs n=30, N=5 sh-[scramble] | MW Rank Sum test | Simple Spikes | 24.488±2.098 vs 20.290±1.648 | p=0.190 |
| S7C |  | MW Rank Sum test | CV1 | 2.565±0.292 vs 2.145±0.314 | p=0.206 |
| S7D |  | Student’s t-test | CV2 | 0.671±0.028 vs 0.789±0.0294 | **p=0.005** |
| S7F |  | MW Rank Sum test | Complex Spikes | 0.175±0.0189 vs 0.217±0.024 | p=0.140 |
| S7G |  | MW Rank Sum test | CV1 | 0.809±0.0259 vs 0.783±0.0464 | p=0.226 |
| S7H |  | Student’s t-test | CV2 | 0.876±0.0286 vs 0.892±0.0352 | p=0.728 |

Table S10. List of statistical tests, p-values and data of Supplementary Figures. Significant p-values are highlighted in bold.

Movie S1.

Tottering with sh-ADRA1D expression does not exhibit stress-induced dystonia.

Movie S2.

Tottering with sh-scramble expression at the onset of stress-induced dystonia displays motor deficiencies.

Movie S3.

Example *in vivo* calcium recording of a homozygous tottering mouse experiencing dystonia after NaCl injection.

Movie S4.

Example *in vivo* calcium recording of a homozygous tottering mouse without dystonia after BMY-7378 injection.
